# LncRNA *JINR1* regulates *miR-216b-5p/*GRP78 and *miR-1-3p/*DDX5 axis to promote JEV infection and cell death

**DOI:** 10.1128/jvi.00066-25

**Published:** 2025-04-24

**Authors:** Shraddha Tripathi, Suryansh Sengar, Anirban Basu, Vivek Sharma

**Affiliations:** 1 Department of Biological Sciences, Birla Institute of Technology and Science, Pilani, Hyderabad Campus 209298, Hyderabad, Telangana, India; 2 National Brain Research Centre 29050, Manesar, Haryana, India; University of North Carolina at Chapel Hill, Chapel Hill, North Carolina, USA

**Keywords:** JEV, WNV, *JINR1*, GRP78, DDX5, *miR-216b-5p*, *miR-1-3p*, RBM10, LINC01518

## Abstract

Japanese encephalitis virus (JEV) infection in the central nervous system (CNS) leads to neuroinflammation and neuronal cell death. Several long non-coding RNAs (lncRNAs) are differentially expressed during viral infection and regulate multiple aspects of viral pathogenesis. Previously, we have shown that JEV/West Nile virus (WNV) infection promotes JEV-induced non-coding RNA 1 (*JINR1*) expression in SH-SY5Y cells, and it interacts with RNA-binding motif protein 10 (RBM10) to enhance cell death and viral replication. In this study, we show that JEV or WNV infection of the SH-SY5Y cells inhibits the expression of microRNAs (miRNAs) *miR-216b-5p* and *miR-1-3p*. These miRNAs bind to the JEV/WNV genome, and their overexpression during JEV/WNV infection reduces viral replication and cell death. Depleting *JINR1* or RBM10 during viral infection prevents the downregulation of *miR-216b-5p* and *miR-1-3p*. In addition, *JINR1* or RBM10 knockdown during JEV/WNV infection enhances the binding of RNA Pol II and H3K4me3 at the promoters of *miR-216b-5p* and *miR-1-3p. JINR1* or RBM10 depletion also prevents the binding of H3K27me3 at the promoters of these miRNAs, suggesting that *JINR1* and RBM10 are involved in their transcription repression. Interestingly*, JINR1* also acts as a competing endogenous RNA (ceRNA) that directly binds to *miR-216b-5p* and *miR-1-3p,* resulting in the upregulation of their targets glucose-regulated protein 78 (GRP78) and DEAD-Box Helicase 5 (DDX5), respectively, which are involved in regulating viral replication. Our findings suggest that *JINR1* uses multiple mechanisms to promote JEV and WNV infection in neuronal cells.

**IMPORTANCE:**

Infection of the central nervous system (CNS) by Japanese encephalitis virus (JEV) or West Nile virus (WNV) leads to neuroinflammation and neuronal cell death. Long non-coding RNAs (lncRNAs) and microRNAs (miRNAs) regulate viral infection by regulating the expression of host genes. However, knowledge about the interplay between lncRNAs and miRNAs during JEN/WNV infection is limited. We show that JEV/WNV infection inhibits the expression of anti-viral host miRNAs *miR-216b-5p* and *miR-1-3p*. These miRNAs inhibit the JEV and WNV replication by directly binding with their genome. *JINR1* and its interacting protein, RBM10, inhibit the transcription of *miR-216b-5p* and *miR-1-3p*. Interestingly, *JINR1* also binds and sequesters *miR-216b-5p* and *miR-1-3p*, resulting in upregulation of their targets GRP78 and DDX5, respectively, which promote viral infection. Our findings suggest that lncRNA *JINR1* is a potential target for developing anti-virals against JEV/WNV infection.

## INTRODUCTION

Japanese encephalitis virus (JEV) and West Nile virus (WNV) are positive-sense, single-stranded RNA viruses that belong to the orthoflavivirus genus of the Flaviviridae family (1). Most orthoflavivirus genus members are dual-host viruses transmitted horizontally between vertebrate hosts and arthropod vectors such as mosquitoes and ticks (2). The clinical spectrum of orthoflavivirus infections is broad and generally divided into two main categories: systemic diseases involving vascular shock syndrome and viral hemorrhagic fevers (VHFs), such as those caused by dengue virus (DENV) and yellow fever virus (YFV), while some orthoflaviviruses such as JEV, WNV, Zika virus (ZIKV), and tick-borne encephalitis virus (TBEV) are neuroinvasive and neurovirulent, capable of causing damage to the central nervous system (CNS) (3–5). A JEV or WNV infection of the CNS results in neuronal cell death and inflammation (6–11). The JEV/WNV infection of neurons is marked by the accumulation of misfolded proteins, exaggerated pro-inflammatory response, and activation of apoptotic machinery, leading to neuronal death (12, 13). Even though vaccines against JEV are available for human use, their administration is not widespread (14–16). Unlike JEV, no vaccine against WNV has been approved for human use (17). Because there are no clinically approved anti-viral drugs for treating JEV/WNV infection, it is imperative to comprehensively understand the biology of these viruses and develop strategies to combat illnesses associated with their infection.

Long non-coding RNAs (lncRNAs) were initially described as regulatory non-coding RNA molecules longer than 200 nucleotides, but they have recently been re-defined to include non-coding RNA exceeding a length of 500 nucleotides (18–21). LncRNAs play integral roles in regulating cellular functions in health and disease by regulating gene expression through RNA–RNA, RNA–DNA, and RNA–protein interactions (22, 23). Several lncRNAs function as competing endogenous RNAs (ceRNAs), binding and sequestering microRNAs (miRNAs) to reduce miRNA-mediated post-transcriptional gene silencing (24, 25). LncRNAs modulate viral replication and host response by regulating gene expression at the epigenetic, transcriptional, and post‐transcriptional levels (26, 27).

Several lncRNAs regulate orthoflavivirus pathogenesis, but there is limited knowledge about how they function and regulate neuronal cell death and neuroinflammation (28, 29). JEV infection increases the expression of lncRNAs NEAT1 and MALAT1 in mice brains and N2A cells, respectively, but their exact function in JEV pathogenesis is unknown (30, 31). JEV infection increases the expression of lncRNAs E52329 and N54010 in mice brains, and they regulate inflammation in microglial cells by activating the MKK4/JNK pathway (32). JEV or WNV infection promotes the expression of lncRNA Gm20559 in mouse microglial cells, and its inhibition reduces the expression of pro-inflammatory cytokines (33). Several lncRNAs are differentially expressed (DE) upon ZIKV infection of human neuroprogenitor cells (34–36). Selinger et al. used an integrative whole transcriptome analysis to identify multiple DE mRNAs, miRNAs, and lncRNAs in human neurons and astrocytes during the TBEV infection (37). However, none of these studies explored the functional role of these lncRNAs in virus pathogenesis. We have recently shown that JEV/WNV infection of SH-SY5Y cells increases the expression of lncRNA *JINR1* (*LINC01518*) and RBM10 protein. *JINR1* and RBM10 interact to promote viral replication, associated neuronal apoptosis, and the expression of NF-κB target genes involved in endoplasmic reticulum (ER) stress and neuroinflammation (38). *JINR1* and RBM10 enhance viral replication in SH-SY5Y cells by increasing GRP78 expression (38).

*JINR1* expression is upregulated in human glaucoma tissues and esophageal squamous cell carcinoma (ESCC) (39, 40). It acts as a ceRNA for *miR-1-3p* in ESCC cells and positively regulates (PIK3CA)/protein kinase B (Akt) pathways (39). *JINR1* promotes proliferation, migration, and autophagy in human tenon capsule fibroblast (HTF) by sponging *miR-216b-5p*, but the target genes of *miR-216b-5p* in these cells remain unknown (40). Given that *JINR1* regulates JEV/WNV infection, we explored the role of *miR-216b-5p* and *miR-1-3p* during JEV and WNV infection.

We show that JEV/WNV infection causes downregulation of *miR-216b-5p* and *miR-1-3p* expression in the SH-SY5Y cells. *miR-216b-5p* and *miR-1-3p* directly bind to the JEV and WNV genome, and their overexpression during JEV/WNV infection reduces viral replication and associated neuronal cell death. *JINR1* and its interacting protein, RBM10, are involved in the inhibition of transcription of *miR-216b-5p* and *miR-1-3p* during viral infection in SH-SY5Y cells. Moreover, we show that *JINR1* sponges *miR-216b-5p* and *miR-1-3p* during viral infection to enhance the expression of their target genes GRP78 and DDX5, respectively, thereby promoting viral infection. Our results reaffirm the pivotal role of *JINR1* in regulating JEV and WNV replication and neuronal cell death.

## MATERIALS AND METHODS

### Cell culture, virus propagation, and infection

SH-SY5Y, Vero, PS, C6/36, and HEK-293T cells were purchased from the National Centre for Cell Science (NCCS), Pune, and were maintained as described previously (38). All cell lines used in this study have been tested and found free from mycoplasma contamination. Vero cells were used to propagate JEV (GP78 strain), and WNV (Eg101 strain) was propagated in C6/36 cells. The viral titer and infection duration are mentioned in the figures.

### Plaque assay analysis for virus production

As described previously, plaque assay for JEV and WNV was carried out in PS and Vero cells, respectively (38). Briefly, 2 × 10^5^ cells were seeded in 6-well tissue culture plates, and the following day, the cells were infected with the serially diluted virus for 2 hours (38). Cells were then rinsed twice with 1× phosphate-buffered saline (PBS) to remove the excess virus and were topped with 2% low-melting agarose and 2× minimum essential medium (MEM) growth media mixture (1:1). After growing for 4 days at 37°C with 5% CO_2_, cells were fixed with 4% formaldehyde at 37°C for 1 hour (38). Cells were stained for 5 minutes at 37°C by adding 0.5% crystal violet solution and were washed thrice with reverse osmosis (RO) water, and then plaques were counted to calculate the concentration of virus in plaque-forming units (PFU) per milliliter (38). The titer was calculated using the following formula (PFU = *N* × DF/*V* [*N*, number of plaques; DF, virus dilution factor; and *V*, volume of the inoculum]) (38).

### Plasmids and cloning

To confirm the interaction between miRNAs and NS5 and 3′ UTR RNA fragments of JEV and WNV, we cloned the 3′ UTR and NS5 genomic region of JEV and WNV in the pmirGLO dual-luciferase miRNA target expression vector (**#**E1330, Promega). Briefly, the total RNA extracted from the JEV/WNV-infected SH-SY5Y cells was used as the template for reverse transcription PCR (RT–PCR). PrimeScript first-strand cDNA kit (**#**6110A, Takara) was used for reverse transcription (RT) reaction with gene-specific primers (0.2 µM) for JEV/WNV 3′ UTR region. The specific viral genome regions were amplified by PCR and cloned into the pmirGLO plasmid at the NheI and SacI sites. To confirm the interaction between miRNAs and *JINR1*, we cloned the full-length *JINR1* into the pmirGLO vector at the NheI and SacI sites. To verify the interaction between *miR-1-3p* and DDX5 3′ UTR, and *miR-216b-5p* and GRP78 3′ UTR, we cloned the *DDX5* 3′ UTR and GRP78 3′ UTR at the 3′ end of the Renilla luciferase gene of the pmiRGLO plasmid (denoted as pmiRGLO-DDX5-3′ UTR and pmiRGLO-GRP78-3′ UTR) at the NheI and SacI sites. The cloning of full-length human *JINR1* (1,856 bp) into the pcDNA3.1 is described earlier (38). The full-length RBM10 construct (pDest26-RBM10) was a kind gift from Prof. Juan Valcárcel, Center for Genomic Regulation, Pompeu Fabra University, Spain (41). The full-length p68/DDX5 (pcDNA-DDX5) construct was a kind gift from Prof. Ralf Janknecht, University of Oklahoma Health Sciences Centre (42). All constructs were verified by Sanger sequencing. The RT and cloning PCR primer sequences are listed in Table S1.

### Site-directed mutagenesis

The Q5 Site-Directed Mutagenesis Kit (#E54S, NEB) was used to generate mutations in *miR-216b-5p*/*miR-1-3p* binding sites in the *JINR1*, GRP78, DDX5, and viral genome fragments (NS5 and 3′ UTR region of JEV/WNV) cloned in pmirGLO vector according to the manufacturer’s instructions. Mutation-specific forward and reverse PCR primers were designed using the NEBaseChanger tool (https://nebasechanger.neb.com/). The primer sequences are provided in Table S1. All mutant constructs were verified by sequencing.

### RNA isolation and real-time PCR

RNA isolation and qRT-PCR were performed as described previously (38). The relative gene expression of each sample was calculated using the 2^−ΔΔ*C*t^ formula. For miRNA expression analysis, RNA was isolated using Quick-RNA Miniprep Plus Kit (**#**R1057, Zymo). The cDNA synthesis was conducted using the mir-X miRNA 1st-Strand Synthesis Kit (**#**638313, Takara). qRT-PCR of miRNAs was carried out using the reverse universal primer and the forward primer specific to *hsa-miR-216b-5p* and *hsa-miR-1-3p*. U6 was used as a normalizing control for miRNA genes. The primer sequences are listed in Table S1.

### Western blot analysis

Protein isolation and Western blotting were carried out as described previously (38, 43, 44). The following primary and secondary antibodies were used: GRP78 (1:5,000, **#**ab21685, Abcam), Cleaved PARP (1:2,000, **#**5625, CST), DDX5 (1:3,000, **#**9877, CST), horseradish peroxidase (HRP)-conjugated anti-rabbit (#PI 2000, Vector Laboratories), and anti-mouse IgG (**#**A16072, Invitrogen). The Western blot images were quantified using ImageJ software for Microsoft Windows (National Institutes of Health, Bethesda, MD, USA).

### Caspase 3/7 assay

A luminometric assay kit for caspase-3/7 (#G8090, Promega) was used to determine the enzymatic activity of caspase-3/7 in virus-infected SH-SY5Y cells transfected with mimic-miR-NS, *miR-216b-5p* mimic, *miR-216b-5p* inhibitor, *miR-1-3p* mimic, *miR-1-3p* inhibitor, and inhibitor-NS as per the manufacturer’s instructions.

### miRNA target analysis

The interaction between miRNAs and JEV/WNV genome was identified using IntaRNA (https://www.rnasociety.org/rnainter/IntaRNA.html) and RNAhybrid (http://bibiserv.techfak.uni-bielefeld.de/rnahybrid/) based on minimum free energy (MFE) and hybridization patterns (45, 46). mRNA targets of *miR-216b-5p/miR-1-3p* were identified using miRWalk (http://mirwalk.umm.uni-heidelberg.de/) and IntaRNA database (47).

### Antisense oligo (ASO)/siRNA transfection

SH-SY5Y cells were reverse transfected with 40 nM of two different ASOs targeting *JINR1* or non-specific ASO-NS using Lipofectamine RNAiMAX (#13778-075, Life Technologies-Invitrogen) as described previously (38). siRNA duplexes against RBM10 (#4390824, Applied Biosystems) and non-specific siRNA that does not target any known mammalian gene (#D-001810-10-20, Dharmacon) were reverse transfected at a final concentration of 40 nM with Lipofectamine RNAiMAX (Invitrogen) as described earlier (38). To overexpress or inhibit *miR-216b-5p/miR-1-3p*, we reverse transfected SH-SY5Y cells with a final concentration of 80 nM of *miR-1-3-p* mimic (#YM00472818-ADA, Qiagen), *miR-216b-5p* mimic (#YM00470958-ADA, Qiagen), mimic-miR-non-specific (#YM00479902-ADA, Qiagen), *miR-1-3p* inhibitor (#YI04100840-ADA, Qiagen), *miR-216b-5p* inhibitor (#YI04101761-ADA, Qiagen), and inhibitor-miR-non-specific (Qiagen, YI00199006-ADA) according to the manufacturer’s instructions.

### Dual-luciferase reporter assay

All dual-luciferase reporter assays were performed in HEK-293T cells using the Dual-Luciferase reporter assay kit (#E1910, Promega), and luminescence was measured using the SpectraMax iD3 Luminometer (Molecular Devices Corporation). Data were normalized to Renilla Luciferase activity. JetPRIME transfection reagent (#101000046, Polyplus) was used for co-transfection experiments, and luciferase readings were taken 36 hours post-transfection according to the manufacturer’s instructions. To confirm the interaction between *miR-216b-5p*, *miR-1-3p*, and NS5 and 3′ UTR fragments of JEV and WNV, cells were co-transfected with pmirGLO-JEV-NS5/pmirGLO-JEV-3′ UTR/pmirGLO-WNV-NS5/pmirGLO-WNV-3′ UTR plasmids along with mimics of either *miR-216b-5p*, *miR-1-3p,* or mimic-miR-NS (non-specific miRNA mimic). The interaction between *miR-216b-5p*/*miR-1-3p* and *JINR1* was confirmed by co-transfecting cells with pmirGLO-lncRNA-*JINR1* reporter plasmid with mimics of either *miR-216b-5p, miR-1-3p,* or mimic-miR-NS. The interaction between GRP78-3′ UTR and *miR-216b-5p* was confirmed in cells co-transfected with pmirGLO-GRP78-3′ UTR and *miR-216b-5p* mimic/mimic-miR-NS. Cells were co-transfected with pmirGLO-DDX5-3′ UTR and *miR-1-3p* mimic/mimic-miR-NS to study the interaction between DDX5-3′ UTR and *miR-1-3p*.

### Chromatin immunoprecipitation (ChIP)

ChIP analysis of cells was performed as described previously (38, 43). The following antibodies were used for IP: RNA Polymerase II (1:100, #2629, CST), Histone H3 trimethylated at Lysine 4 (1:100, #9751, CST), Histone H3 trimethylated at Lysine 27 (1:100, #9733, CST), anti-rabbit IgG (#2729, CST), and anti-mouse IgG (#5415, CST). The binding of RNA-Pol-II/H3K4me3/H3K27me3 at the promoters of *miR-216b-5p*/*miR-1-3p* was measured using qRT-PCR. The primers specific to the *miR-216b-5p*/*miR-1-3p* promoter used for ChIP-qRT-PCR analysis are listed in Table S1.

### UV cross-linked Ago2 RNA immunoprecipitation (UV Ago2 RIP)

To assess the binding of *JINR1*, GRP78, DDX5, and viral genomes to Ago2 upon miRNA overexpression, UV Ago2 RIP was performed as described previously with minor modifications (48, 49). Briefly, 5 × 10^6^ SHSY-5Y cells were transfected with miR-NS/*miR-216b-5p* and mimic/*miR-1-3p* mimics, and were infected with JEV/WNV (multiplicity of infection [MOI] 5). Thirty-six hours post-infection (hpi), Ago2-bound RNA complexes in cells were cross-linked using 254 nm UV light (450 mJ/cm^2^ in a UV crosslinker CL3000, Analytik Jena). Cross-linked cells were washed twice with ice-cold 1× PBS and were snap-frozen in 100 µL of lysis buffer (10 mM Hepes, pH 7.4, 100 mM KCl, 5 mM MgCl_2_, 0.5% NP-40, 1 mM DTT), supplemented with protease inhibitor (#P8340, Sigma) and RNasin (#N2611, Promega). The snap-frozen pellets were resuspended in 900 µL of ice-cold lysis buffer and were sonicated for 15 minutes in a Bioruptor (Diagenode) set on high (10” ON/40” OFF). Lysates were cleared by centrifugation at 14,000 rpm for 20 minutes. The sonicated lysate was split into equal aliquots, made up to a total volume of 1.5 mL with IP buffer (1% Triton X-100, 0.1% DOC, 1× TE, protease inhibitor, and RNasin), and 5% of the sample was taken out as input. Fifty microliters of the Dynabeads Protein G (#10004D, Invitrogen), anti-Ago2 (#MABE253, Millipore), or IgG antibodies was added and incubated overnight at 4°C. The next day, the beads-bound RNA complexes were washed five times with NT2 buffer (50 mM Tris-HCl, pH 7.5, 150 mM NaCl2, 1 mM MgCl2, 0.05% NP-40, supplemented with protease inhibitor and RNasin) and were resuspended in 100 µL of NT2 buffer. To reverse the cross-linking, the NT2 buffer containing RNA was supplemented with 2 µL proteinase K (NEB), made up to a total volume of 300 µL, and incubated for 2 hours at 65°C. After reverse cross-linking, RNA was extracted using the NucleoSpin RNA kit (MN 740955.50) as per the manufacturer’s instructions. The cDNA synthesis was conducted using the PrimeScript first-strand cDNA kit (#6110A, Takara). qRT-PCR was performed in triplicate with 0.5 µL cDNA using SYBR Green PCR Kit (#RR820A, Takara) on a LightCycler 480 Real-Time PCR System. The results are expressed as fold enrichment over input and normalized to MI/JEV/WNV IgG.

### Statistical analysis

Results are presented as mean ± standard error of the mean (SEM), unless otherwise stated. We used paired Student’s *t*-test for comparisons between two experimental groups. Additional statistical test information is described in the figure legends. *P* < 0.05 was considered statistically significant.

## RESULTS

### JEV/WNV infection results in the downregulation of *miR-1-3p* and *miR-216b-5p* expression in SH-SY5Y cells

**Fig 1:**
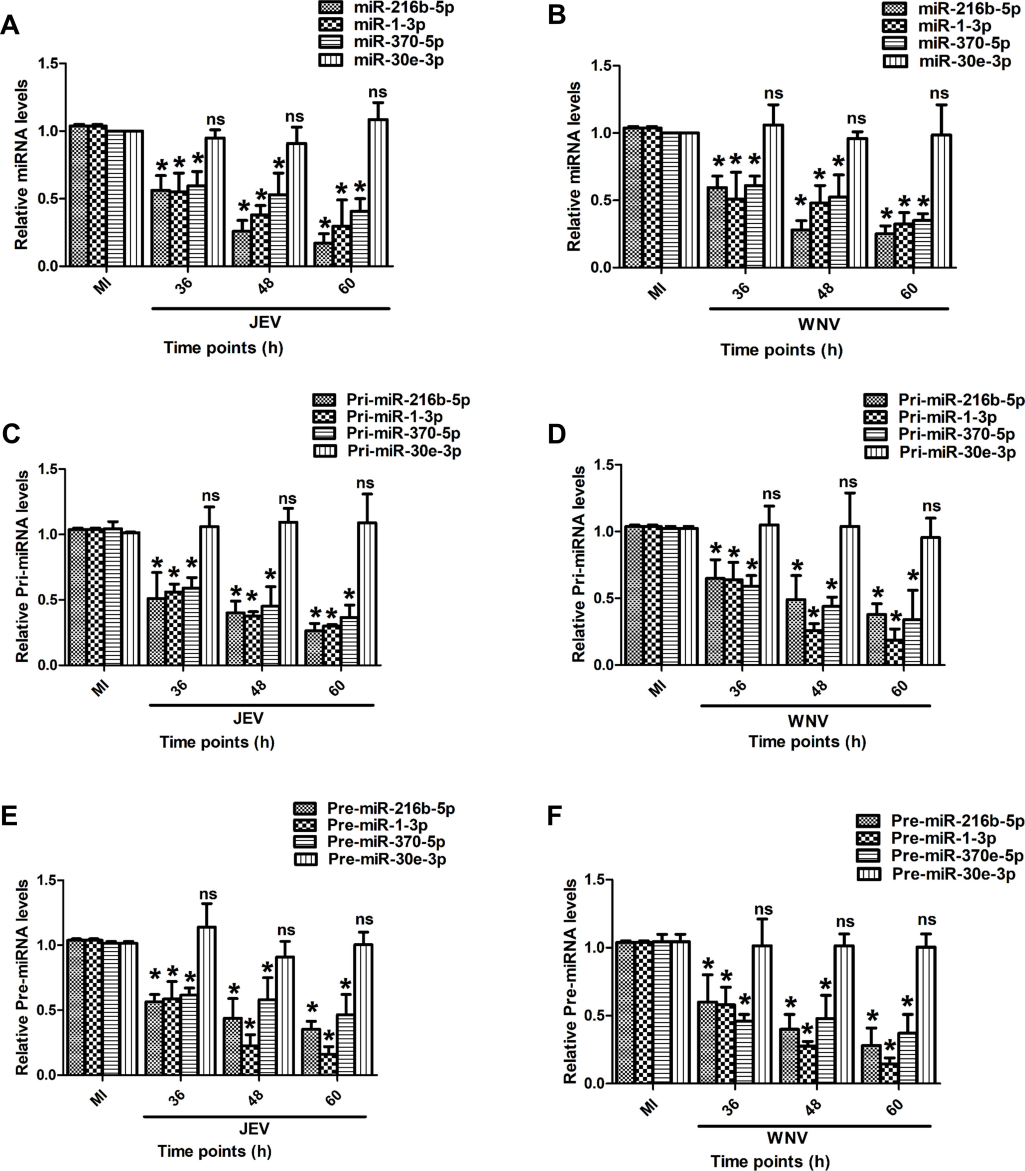
JEV/WNV infection inhibits *miR-216b-5p and miR-1-3p* expression in SH-SY5Y cells. (**A**) Time course analysis of the mature form of *miR-216b-5p, miR-1-3p*, *miR-370-5p*, and *miR-30e-3p* in SH-SY5Y cells at indicated time points during JEV infection. (**B**) Time course analysis of the mature form of *miR-216b-5p, miR-1-3p*, *miR-370-5p*, and *miR-30e-3p* in SH-SY5Y cells at indicated time points during WNV infection. (**C**) Time course analysis of *Pri*-*miR-216b-5p, Pri*-*miR-1-3p, Pri*-*miR-370-5p,* and *Pri-miR-30e-3p* in SH-SY5Y cells at indicated time points during JEV infection. (**D**) Time course analysis of *Pri*-*miR-216b-5p, Pri*-*miR-1-3p, Pri*-*miR-370-5p,* and *Pri-miR-30e-3p* in SH-SY5Y cells at indicated time points during WNV infection. (**E**) Time course analysis of *Pre*-*miR-216b-5p, Pre-miR-1-3p, Pre-miR-370-5p,* and *Pre-miR-30e-3p* in SH-SY5Y cells at indicated time points during JEV infection. (**F**) Time course analysis of *Pre*-*miR-216b-5p, Pre-miR-1-3p, Pre-miR-370-5p,* and *Pre-miR-30e-3p* in SH-SY5Y cells at indicated time points during WNV infection. Data information: error bars represent the mean ± SEM from three independent experiments. Statistical comparisons between groups were made using the Student’s *t*-test. (For A–F) qRT-PCR analysis of indicated transcript upon JEV/WNV infection in SH-SY5Y cells. *Significant change compared to the corresponding MI. ns represents non-significant change compared to the corresponding MI.

Most lncRNAs execute their cellular functions by interacting with proteins and/or miRNAs (19). We have shown that lncRNA *JINR1* interacts with RBM10 to promote JEV and WNV replication and neuronal cell death (38). As *JINR1* also interacts with *miR-1-3p* and *miR-216b-5p* (39, 40), we asked if *miR-1-3p* and *miR-216b-5p* have any role in JEV/WNV infection in SH-SY5Y cells. As host miRNAs can bind to RNA viruses and regulate their replication (50), we first checked if *miR-216b-5p* and *miR-1-3p* have any binding sites in the JEV and WNV genome. Computational screening of the JEV/WNV genome for potential *miR-216b-5p* and *miR-1-3p* binding sites using the IntaRNA and RNAhybrid program revealed multiple *miR-1-3p* and *miR-216b-5p* binding sites in the genome of the GP78 strain of JEV and the Eg101 strain of WNV (Tables 2 and 3) (45, 51). Therefore, we evaluated the impact of JEV/WNV infection on *miR-216b-5p* and *miR-1-3p* expression in SH-SY5Y cells. To this end, we performed a time-dependent expression analysis of the mature forms of *miR-216b-5p* and *miR-1-3p* during JEV and WNV infection in SH-SY5Y cells. Interestingly, the levels of mature *miR-216b-5p* and *miR-1-3p* transcripts were significantly downregulated upon JEV/WNV infection in a time-dependent manner in SH-SY5Y cells (Fig. 1A and B).

*miR-370-5p* expression is downregulated during JEV/WNV infection (51, 52), and it was used as a positive control. As expected, its expression is downregulated during JEV or WNV infection in SH-SY5Y cells (Fig. 1A and B). Mature *miR-30e-3p* expression is stable during WNV infection and was used as a negative control (52). The expression of *miR-30e-3p* remained unchanged during JEV/WNV infection in SH-SY5Y cells (Fig. 1A and B). These results confirm that JEV/WNV infection of SH-SY5Y results in the downregulation of mature forms of *miR-216b-5p* and *miR-1-3p*.

### *miR-216b-5p* and *miR-1-3p* downregulation during JEV/WNV infection is due to transcription inhibition

The decrease in the expression of mature forms of *miR-1-3p* and *miR-216b-5p* during JEV and WNV infection can be due to either transcription inhibition or reduced miRNA processing. To understand the mechanism of *miR-1-3p* and *miR-216b-5p* downregulation upon viral infection in SH-SY5Y cells, we measured the expression of primary and precursor transcripts of *miR-216b-5p* and *miR-1-3p* during JEV/WNV infection. The expression of primary *miR-216b-5p* (*pri*-*miR-216b-5p*) and primary miR-1-3p (*pri*-*miR-1-3p*) transcripts was also downregulated in a time-dependent manner following JEV/WNV infection (Fig. 1C and D). Similarly, the expression of *miR-216b-5p* precursor (*pre*-*miR-216b-5p*) and *miR-1-3p* precursor (*pre*-*miR-1-3p*) transcripts was also downregulated during JEV/WNV infection (Fig. 1E and F). The expression of positive control *miR-370-5p* primary transcript (*pri*-*miR-370-5p*) and precursor transcript (*pre*-*miR-370-5p*) was also downregulated after JEV/WNV infection in SH-SY5Y cells. The expression of primary *miR-30e-3p* (*pri*-*miR-30e-3p*) and precursor *miR-30e-3p* (*pre-miR-30e-3p*) transcripts remained unchanged following JEV/WNV infection in SH-SY5Y cells (Fig. 1C through F). The reduction in the levels of the primary transcripts of *miR-216b-5p* and *miR-1-3p* suggests that the downregulation of *miR-216b-5p* and *miR-1-3p* occurs during transcription upon JEV/WNV infection.

**Fig 2:**
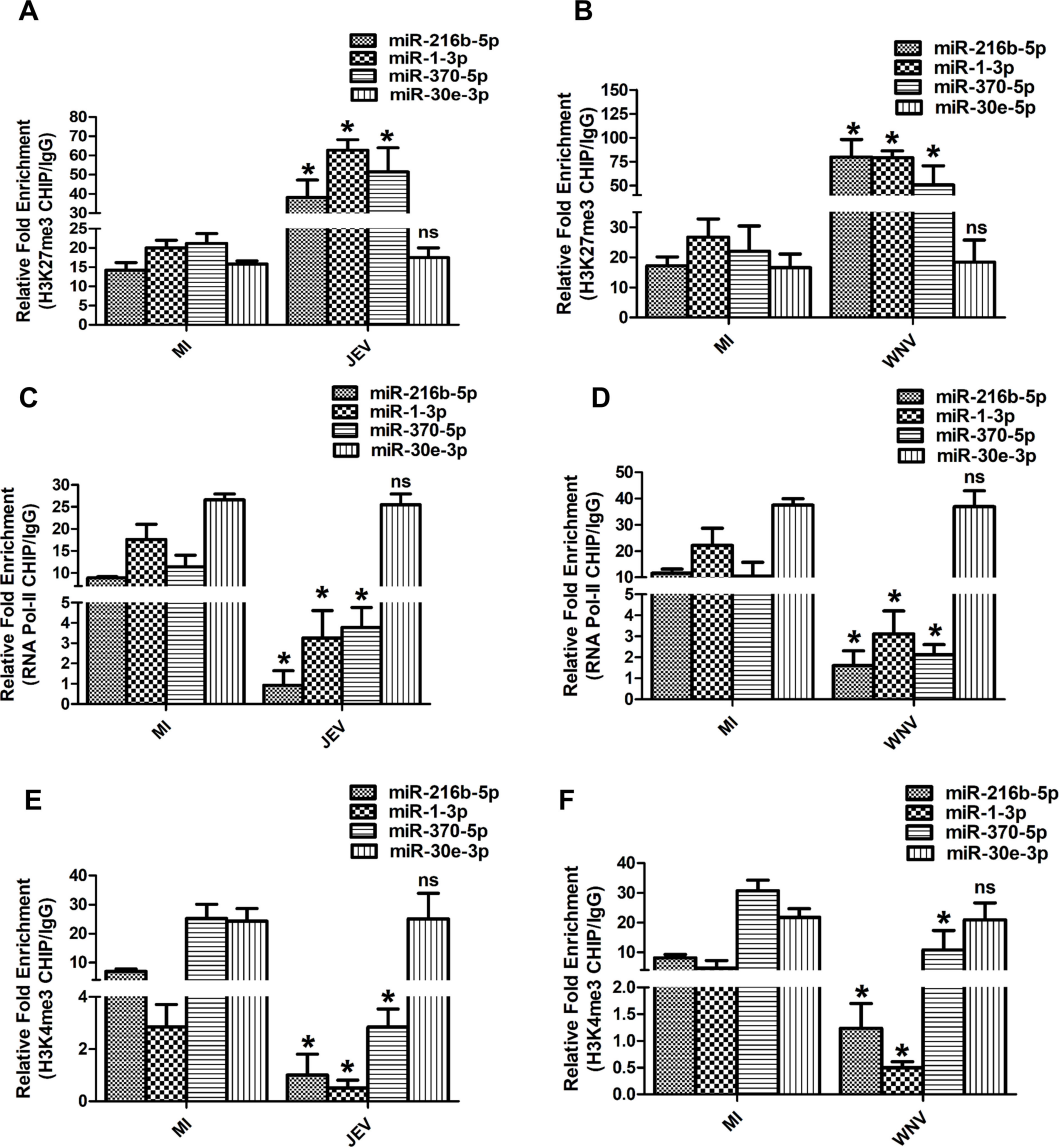
JEV/WNV infection results in the transcription inhibition of *miR-216b-5p* and *miR-1-3p* in SH-SY5Y cells. (**A**) Effect of JEV infection on H3K27me3 recruitment at the promoters of *miR-216b-5p, miR-1-3p, miR-370-5p,* and *miR-30e-3p*. Relative enrichment of H3K27me3 at the *miR-216b-5p, miR-1-3p, miR-370-5p,* and *miR-30e-3p* promoter region in MI or JEV-infected SH-SY5Y cells determined by ChIP-qRT-PCR at 36 hpi. (**B**) Effect of WNV infection on H3K27me3 recruitment at the promoters of *miR-216b-5p, miR-1-3p, miR-370-5p,* and *miR-30e-3p*. Relative enrichment of H3K27me3 at the *miR-216b-5p, miR-1-3p, miR-370-5p,* and *miR-30e-3p* promoter region in MI or WNV-infected SH-SY5Y cells determined by ChIP-qRT-PCR at 36 hpi. (**C**) Effect of JEV infection on RNA Pol II recruitment at the promoters of *miR-216b-5p, miR-1-3p, miR-370-5p,* and *miR-30e-3p*. Relative enrichment of RNA Pol II at the *miR-216b-5p, miR-1-3p, miR-370-5p,* and *miR-30e-3p* promoter region in MI or JEV-infected SH-SY5Y cells determined by ChIP-qRT-PCR at 36 hpi. (**D**) Effect of WNV infection on RNA Pol II recruitment at the promoters of *miR-216b-5p, miR-1-3p, miR-370-5p,* and *miR-30e-3p*. Relative enrichment of RNA Pol II at the *miR-216b-5p, miR-1-3p, miR-370-5p,* and *miR-30e-3p* promoter region in MI or WNV-infected SH-SY5Y cells determined by ChIP-qRT-PCR at 36 hpi. (**E**) Effect of JEV infection on H3K4me3 recruitment at the promoters of *miR-216b-5p, miR-1-3p, miR-370-5p,* and *miR-30e-3p*. Relative enrichment of H3K4me3 at the *miR-216b-5p, miR-1-3p, miR-370-5p,* and *miR-30e-3p* promoter region in MI or JEV-infected SH-SY5Y cells determined by ChIP-qRT-PCR at 36 hpi. (**F**) Effect of WNV infection on H3K4me3 recruitment at the promoters of *miR-216b-5p, miR-1-3p, miR-370-5p,* and *miR-30e-3p*. Relative enrichment of H3K4me3 at the *miR-216b-5p, miR-1-3p, miR-370-5p,* and *miR-30e-3p* promoter region in MI or WNV-infected SH-SY5Y cells determined by ChIP-qRT-PCR at 36 hpi. Data information: error bars represent the mean ± SEM from three independent experiments. Statistical comparisons between groups were made using the Student’s *t*-test. (For A–F) DNA samples were analyzed by qRT-PCR. Enrichment values are relative to MI-IgG. *Significant change compared to the corresponding MI. ns represents non-significant change compared to the corresponding MI.

To confirm the transcriptional silencing of these miRNAs during JEV/WNV infection, we performed ChIP qRT-PCR to assess the binding of H3K27me3, RNA Pol-II, and H3K4me3 at the promoters of *miR-216b-5p*, *miR-1-3p, miR-30e-3p,* and *miR-370-5p* during JEV/WNV infection. JEV/WNV infection resulted in significantly enhanced binding of the repressive H3K27me3 mark at the promoters of *miR-216b-5p, miR-1-3p,* and *miR-370-5p* in comparison to mock-infected (MI) cells (Fig. 2A and B). Moreover, the binding of RNA Pol-II and H3K4me3 marks at the promoters of *miR-216b-5p, miR-1-3p,* and *miR-370-5p* was significantly less in JEV/WNV-infected cells than in MI cells (Fig. 2C through F).

Moreover, there was no significant change in the binding of H3K27me3, RNA Pol-II, and H3K4me3 marks at the promoter of *miR-30e-3p* (Fig. 2C through F). In addition, we did not observe any significant change in the mRNA expression of miRNA processing enzymes Drosha, Dicer, and DGCR8 upon JEV/WNV infection in SH-SY5Y cells (Fig. S1). Collectively, these results indicate that the inhibition of *miR-216b-5p, miR-1-3p,* and *miR-370-5p* expression during JEV or WNV infection is likely due to transcription inhibition and not because of changes in miRNA processing.

### *miR-216b-5p* and *miR-1-3p* inhibit JEV/WNV replication by directly binding to the viral genome

**Fig 3:**
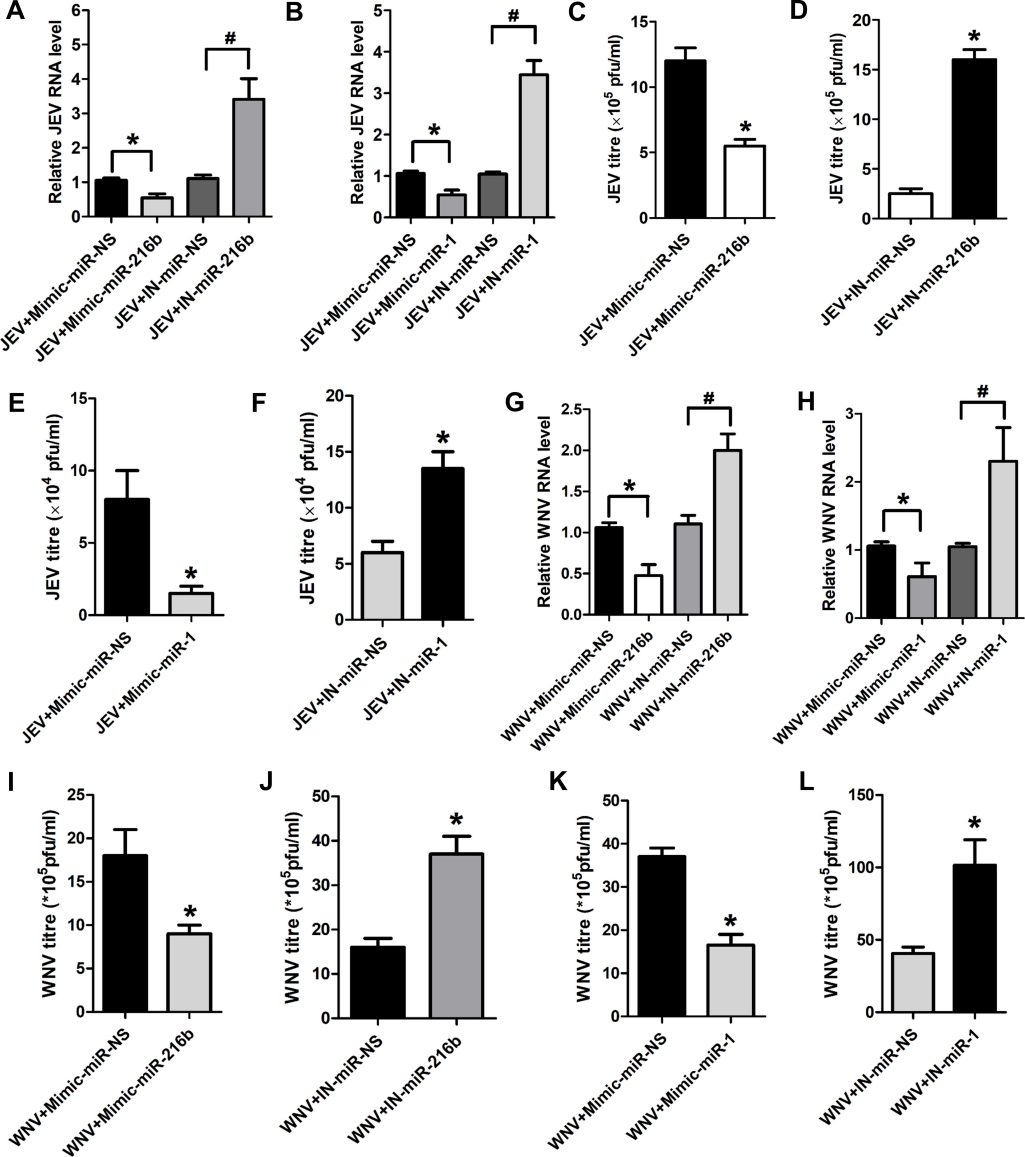
*miR-216b-5p* and *miR-1-3p* negatively regulate JEV/WNV replication in SH-SY5Y cells. (**A**) *miR-216b-5p* inhibits JEV replication in SH-SY5Y cells. Cells were transfected with either mimic/inhibitor of *miR-216b-5p* or mimic-miR-NS/IN-miR-NS (non-specific mimic, non-specific inhibitor) and infected with JEV. Viral replication was determined by quantifying the intracellular levels of JEV RNA using qRT-PCR at 48 hpi. (**B**) *miR-1-3p* inhibits JEV replication in SH-SY5Y cells. Cells were transfected with either mimic/inhibitor of *miR-1-3p* or mimic-miR-NS/IN-miR-NS and infected with JEV. Viral replication was determined by quantifying the intracellular levels of JEV RNA using qRT-PCR at 48 hpi. (**C**) *miR-216b-5p* overexpression decreases JEV titer. Quantification of viral titer upon transfection with mimic miR-216b-5p or mimic-miR-NS during JEV infection. (**D**) *miR-216b-5p* silencing increases JEV titer. Quantification of viral titer upon transfection with IN-miR-216b-5p or IN-miR-NS during JEV infection. (**E**) *miR-1-3p* overexpression decreases JEV titer. Quantification of viral titer upon transfection with mimic-miR-1-3p or mimic-miR-NS during JEV infection. (**F**) *miR-1-3p* silencing increases JEV titer. Quantification of viral titer upon transfection with IN-miR-1-3p or IN-miR-NS during JEV infection. (**F**) *miR-216b-5p* inhibits WNV replication in SH-SY5Y cells. Cells were transfected with either mimic/inhibitor of *miR-216b-5p* or mimic-miR-NS/IN-miR-NS and infected with WNV. Viral replication was determined by quantifying the intracellular levels of WNV RNA using qRT-PCR at 48 hpi. (**G**) *miR-1-3p* inhibits WNV replication in SH-SY5Y cells. Cells were transfected with either mimic/inhibitor of *miR-1-3p* or mimic-miR-NS/IN-miR-NS and infected with WNV. Viral replication was determined by quantifying the intracellular levels of WNV RNA using qRT-PCR at 48 hpi. (**H**) *miR-216b-5p* overexpression decreases WNV titer. Quantification of viral titer upon transfection with mimic-miR-216b-5p or mimic-miR-NS during WNV infection. (**I**) *miR-216b-5p* silencing increases WNV titer. Quantification of viral titer upon transfection with IN-miR-216b-5p or IN-miR-NS during WNV infection. (**J**) *miR-1-3p* overexpression decreases WNV titer. Quantification of viral titer upon transfection with mimic-miR-1-3p or mimic-miR-NS during WNV infection. (**K**) *miR-1-3p* silencing increases WNV titer. Quantification of viral titer upon transfection with IN-miR-1-3p or IN-miR-NS during WNV infection. Data information: error bars represent the mean ± SEM from three independent experiments. Statistical comparisons between groups were made using the Student’s *t*-test. (For A, B, **G, and H**) RNA samples were analyzed by qRT-PCR. (For A and **B**) *Significant change compared to respective JEV+Mimic-miR-NS; ^#^significant change compared to respective JEV+IN-miR-NS. (For C, **E, I, and K**) *Significant change compared to corresponding JEV+Mimic-miR-NS/WNV+Mimic-miR-NS. (For D, **F, J, and L**) *Significant change compared to corresponding JEV+IN-miR-NS/WNV+IN-miR-NS. (For G and **H**) *Significant change compared to respective WNV+Mimic-miR-NS; ^#^significant change compared to respective WNV+IN-miR-NS.

Because *miR-216b-5p* and *miR-1-3p* expression is downregulated during JEV/WNV infection, and they have binding sites on the JEV and WNV genome, we evaluated the impact of *miR-216b-5p*/*miR-1-3p* overexpression and depletion on JEV and WNV replication. SH-SY5Y cells transfected with mimics of either *miR-216b-5p* or *miR-1-3p* (mimic-miR-216b/mimic-miR-1) and infected with JEV had ~50% lower intracellular JEV RNA levels compared to JEV-infected SH-SY5Y cells transfected with mimic-miR-non-specific (Mimic-miR-NS) (Fig. 3A and B). Moreover, *miR-216b-5p* or *miR-1-3p* inhibition using miRNA inhibitors (IN-miR-216b/IN-miR-1) during JEV infection of SH-SY5Y cells had an approximately threefold increase in the expression of intracellular JEV-RNA levels in comparison to JEV-infected cells transfected with inhibitor-miR-nonspecific (IN-miR-NS) (Fig. 3A and B). JEV plaque assay from the culture supernatant of JEV-infected SH-SY5Y cells transfected with *miR-216b-5p/miR-1-3p* mimics or inhibitors confirmed that *miR-216b-5p* and *miR-1-3p* inhibit JEV replication (Fig. 3C through F). Similarly, WNV-infected SH-SY5Y cells overexpressing *miR-216b-5p* or *miR-1-3p* had ~40% less WNV RNA levels compared to WNV-infected SH-SY5Y cells transfected with mimic-miR-NS (Fig. 3G and H). Moreover, *miR-216b-5p* or *miR-1-3p* inhibition during WNV infection of SH-SY5Y cells significantly increases the expression of intracellular WNV-RNA levels compared to control cells (Fig. 3G and H). Determination of WNV titer by plaque assay from the culture supernatant of SH-SY5Y cells transfected with mimics or inhibitors of *miR-216b-5p*/*miR-1-3p* also confirmed that *miR-216b-5p* and *miR-1-3p* negatively regulate WNV replication (Fig. 3I through L).

**Fig 4:**
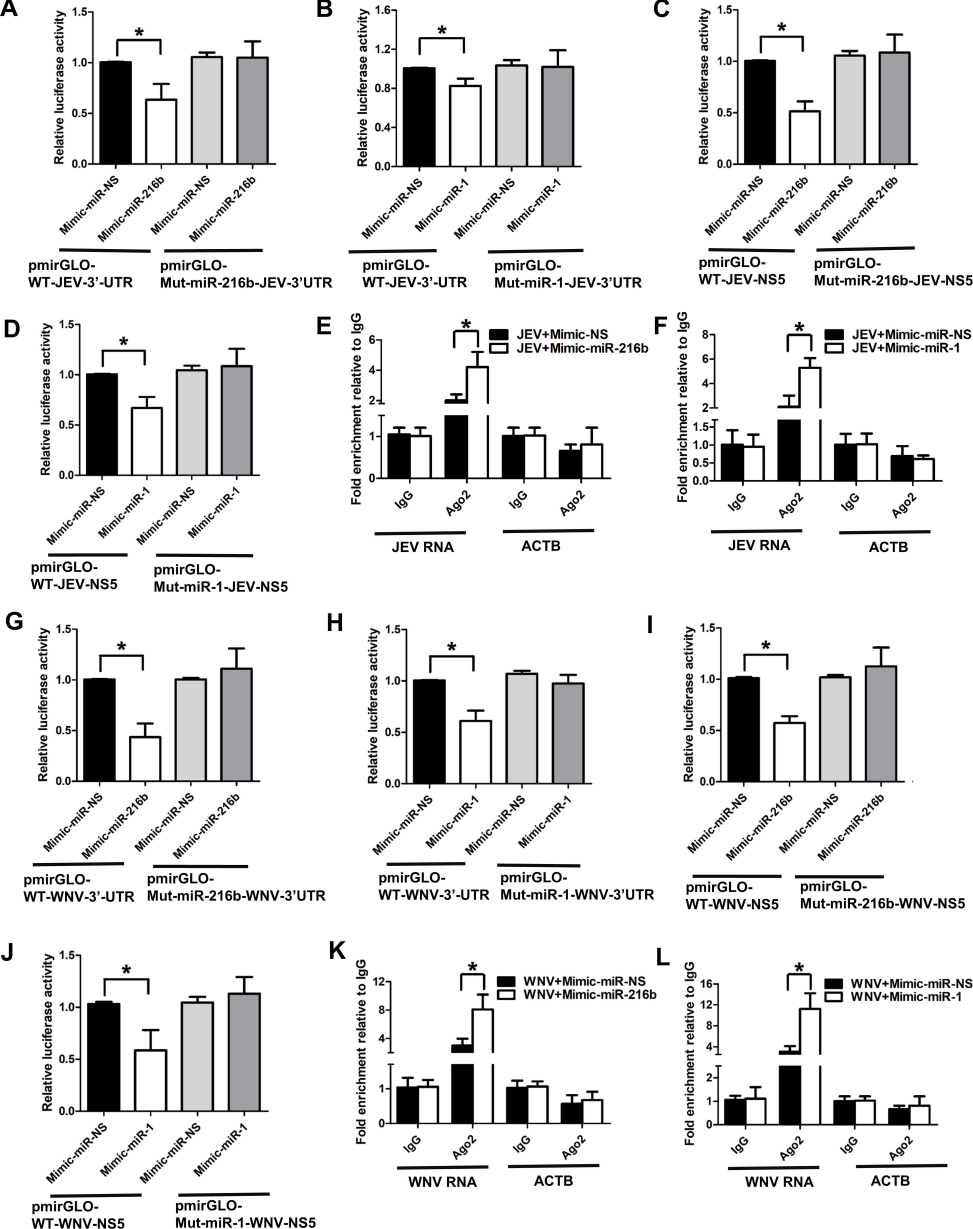
*miR-216b-5p* and *miR-1-3p* interact with the JEV/WNV genome. (**A**) *miR-216b-5p* binds with the JEV 3′ UTR region. Dual luciferase activity was measured in HEK-293T cells co-transfected with the pmiRGLO-WT-JEV-3′ UTR or pmiRGLO-mut-miR-216b-JEV-3′ UTR construct and mimics of either *miR-216b-5p* or miR-NS. (**B**) *miR-1-3p* binds with the JEV 3′ UTR region. Dual luciferase activity was measured in HEK-293T cells co-transfected with the pmiRGLO-WT-JEV-3′ UTR or pmiRGLO-mut-miR-1-3p-JEV-3′ UTR construct and mimics of either *miR-1-3p* or miR-NS. (**C**) *miR-216b-5p* binds with the JEV NS5 region. Dual luciferase activity was measured in HEK-293T cells co-transfected with the pmiRGLO-WT-JEV-NS5 or pmiRGLO-mut-miR-216b-JEV-NS5 construct and mimics of either *miR-216b-5p* or miR-NS. (**D**) *miR-1-3p* binds with the JEV NS5 region. Dual luciferase activity was measured in HEK-293T cells co-transfected with the pmiRGLO-WT-JEV-NS5 or pmiRGLO-mut-miR-1-3p-JEV-NS5 construct and mimics of either *miR-1-3p* or miR-NS. (**E**) *miR-216b-5p* overexpression promotes the binding of JEV RNA with Ago2 during JEV infection in SH-SY5Y cells. Anti-Ago2 RIP was performed in SH-SY5Y cells transiently overexpressing *miR-216b-5p* or mimic-NS and infected with JEV, followed by qRT-PCR to detect JEV RNA and ACTB associated with Ago2. The data shown are normalized to input and represented as fold enrichment relative to JEV+Mimic-miR-NS-IgG IP. Values represent the mean ± SEM from three independent experiments. (**F**) *miR-1-3p* overexpression promotes the binding of JEV RNA with Ago2 during JEV infection in SH-SY5Y cells. Anti-Ago2 RIP was performed in SH-SY5Y cells transiently overexpressing *miR-1-3p* or mimic-NS infected and infected with JEV, followed by qRT-PCR to detect JEV RNA and ACTB associated with Ago2. The data shown are normalized to input and represented as fold enrichment relative to JEV+Mimic-miR-NS-IgG IP. Values represent the mean ± SEM from three independent experiments. (**G**) *miR-216b-5p* binds with the WNV-3′ UTR region. Dual luciferase activity was measured in HEK-293T cells co-transfected with the pmiRGLO-WT-WNV-3′ UTR or pmiRGLO-mut-miR-216b-WNV-3′ UTR construct and mimics of either *miR-216b-5p* or miR-NS. (**H**) *miR-1-3p* binds with the WNV-3′ UTR region. Dual luciferase activity was measured in HEK-293T cells co-transfected with the pmiRGLO-WT-WNV-3′ UTR or pmiRGLO-mut-miR-1-WNV-3′ UTR construct and mimics of either *miR-1-3p* or miR-NS. (**I**) *miR-216b-5p* binds with the WNV-NS5 region. Dual luciferase activity was measured in HEK-293T cells co-transfected with the pmiRGLO-WT-WNV-NS5 or pmiRGLO-mut-miR-216b-WNV-NS5 construct and mimics of either *miR-216b-5p* or miR-NS. (**J**) *miR-1-3p* binds with the WNV-NS5 region. Dual luciferase activity was measured in HEK-293T cells co-transfected with the pmiRGLO-WT-WNV-NS5 or pmiRGLO-mut-miR-1-WNV-NS5 construct and mimics of either *miR-1-3p* or miR-NS. (**K**) *miR-216-5p* overexpression promotes the binding of WNV RNA with Ago2 during WNV infection in SH-SY5Y cells. Anti-Ago2 RIP was performed in SH-SY5Y cells transiently overexpressing *miR-216b-5p* or mimic-NS and infected with WNV, followed by qRT-PCR to detect WNV RNA and ACTB associated with Ago2. The data shown are normalized to input and represented as fold enrichment relative to WNV+Mimic-miR-NS-IgG IP. Values represent the mean ± SEM from three independent experiments. (**L**) *miR-1-3p* overexpression promotes the binding of WNV RNA with Ago2 during WNV infection in SH-SY5Y cells. Anti-Ago2 RIP was performed in SH-SY5Y cells transiently overexpressing *miR-1-3p* or mimic-NS and infected with WNV, followed by qRT-PCR to detect WNV RNA and ACTB associated with Ago2. The data shown are normalized to input and represented as fold enrichment relative to JEV+Mimic-miR-NS-IgG IP. Values represent the mean ± SEM from three independent experiments. Data information: error bars represent the mean ± SEM from three independent experiments. Statistical comparisons between groups were made using the Student’s *t*-test. Luminescence signals were measured at 36 hours post-transfection. (For A–D and G–J) *Significant change compared to mimic-miR-NS. (For C, D, I–L) *Significant change compared to JEV+Mimic-miR-NS-IgG-IP/WNV+Mimic-miR-NS-IgG-IP.

*miR-1-3p* and *miR-216b-5p* negatively regulate JEV/WNV replication. Bioinformatics analysis revealed multiple *miR-1-3p* and *miR-216b-5p* binding sites in the NS5 and 3′ UTR region of the JEV/WNV genome (Tables S2 and S3). Therefore, we confirmed the direct binding of these miRNAs to the NS5 and 3′ UTR fragments of the viral genome by performing the dual luciferase reporter assay between viral RNA fragments and miRNAs. To this end, we cloned the wild-type 3′ UTR and NS5 RNA fragments of the JEV and WNV genome downstream of the firefly luciferase gene in the pmirGLO vector. We also generated JEV and WNV NS5 and 3′ UTR viral fragments with mutations in *miR-1-3p*/*miR-216b-5p* binding sites. Cells co-transfected with wild-type JEV 3′ UTR RNA fragment luciferase reporter construct (pmiR-GLO-WT-JEV-3′ UTR) and *miR-216b-5p* mimic had ~37% less luciferase activity than cells co-transfected with mimic-miR-NS and pmiR-GLO-WT-JEV-3′ UTR (Fig. 4A; Fig. S2A). As expected, cells co-transfected with miR-216b-binding site mutant JEV-3′-UTR RNA fragment luciferase reporter construct (pmiR-GLO-mut-miR-216-JEV-3′ UTR) and *miR-216b-5p* mimic had no decrease in the luciferase activity in comparison to cells co-transfected with mimic-miR-NS and pmiR-GLO-WT-JEV-3′ UTR luciferase reporter construct (Fig. 4A; Fig. S2A).

*miR-1-3p* overexpressing cells transfected with pmiR-GLO-WT-JEV-3′ UTR reporter constructs had ~17% less reporter activity in comparison to mimic-miR-NS overexpressing cells transfected with pmiR-GLO-WT-JEV-3′ UTR (Fig. 4B; Fig. S2B). The reporter activity of *Mir-1-3p* overexpression in cells transfected with the miR-1-binding site mutant JEV-3′-UTR RNA fragment (pmiRGLO-mut-miR-1-JEV-3′ UTR) luciferase reporter vector and cells co-transfected with mimic-miR-NS and pmiR-GLO-WT-JEV-3′ UTR luciferase reporter construct was comparable (Fig. 4B; Fig. S2B).

Cells co-transfected with *miR-216b-5p* mimic and luciferase reporter construct with JEV NS5 RNA fragment (pmiR-GLO-WT-JEV-NS5) had ~49% less reporter activity than cells co-transfected with mimic-miR-NS and pmiR-GLO-WT-JEV-NS5 (Fig. 4C; Fig. S2C). Cells co-transfected with *miR-216b-5p* mimic and luciferase reporter construct with miR-216-binding site mutant JEV NS5 RNA fragment (pmiRGLO-mut-miR-216-JEV-NS5) had no significant change in reporter activity in comparison to cells co-transfected with mimic-miR-NS and pmiR-GLO-WT-JEV-NS5 luciferase reporter construct (Fig. 4C; Fig. S2C). About 33% less reporter activity was observed in cells transfected with a mimic of *miR-1-3p* and pmiR-GLO-WT-JEV-NS5 luciferase reporter construct compared to cells overexpressing mimic-miR-NS and pmiR-GLO-WT-JEV-NS5 luciferase reporter construct (Fig. 4D; Fig. S2D). Moreover, *miR-1-3p* overexpression in cells transfected with luciferase reporter constructs with miR-1-binding site mutant JEV NS5 RNA fragments (pmiRGLO-mut-miR-1-JEV-NS5) showed no significant influence on the luciferase reporter activity compared to cells overexpressing mimic-miR-NS and pmiR-GLO-WT-JEV-NS5 luciferase reporter construct (Fig. 4D; Fig. S2D).

In order to confirm if the binding of *miR-216b-5p* and *miR-1-3p* to viral genome results in increased binding of JEV and WNV RNA to Ago2, we performed UV cross-linked Ago2 RNA immunoprecipitation (RIP) upon *miR-216b-5p*/*miR-1-3p* overexpression in JEV/WNV-infected SH-SY5Y cells. RIP analysis indicated that cells transfected with mimic-*miR-216b-5p* had ~3.45-fold more binding of JEV RNA to Ago2 compared to JEV-infected cells transfected with mimic-miR-NS (Fig. 4E). Similarly, *miR-1-3p* mimic-transfected cells had significantly higher binding of JEV RNA to Ago2 than cells transfected with mimic-miR-NS (Fig. 4F).

In addition, reporter assays show that *miR-216b-5p* overexpression resulted in ~56% less pmir-GLO-WT-WNV-3′ UTR reporter activity in comparison to mimic-miR-NS overexpression (Fig. 4G; Fig. S2E). Similarly, transfection of *miR-1-3p* mimics led to ~37% reduction in the pmirGLO-WT-WNV-3′ UTR luciferase activity in comparison to cells transfected with mimic-miR-NS (Fig. 4H; Fig. S2F). As expected, no appreciable decrease in luciferase reporter activity was seen in cells co-transfected with mimic-miR-216b and pmiR-GLO-mut-miR-216-WNV-3′ UTR compared to the control (Fig. 4G; Fig. S2E). There was no significant difference in pmiR-GLO-mut-miR-1-WNV-3′ UTR luciferase activity upon overexpression of mimic-miR-NS or *miR-1-3p* mimic (Fig. 4H; Fig. S2F).

Overexpression of *miR-216b-5p* resulted in ~43% less pmirGLO-WT-WNV NS5 luciferase activity than overexpression of mimic-miR-NS (Fig. 4I; Fig. S2G). Similarly, *miR-1-3p* overexpressing cells had significantly less pmirGLO-WT-WNV-NS5 luciferase activity than mimic-miR-NS overexpressing cells (Fig. 4J; Fig. S2H). The decrease in luciferase reporter activity during miRNA overexpression was lost upon mutation of the *miR-216b-5p/miR-1-3p* binding site in the NS5 region of WNV (pmiR-GLO-mut-miR-216-WNV-NS5/pmiR-GLO-mut-miR-1-WNV-NS5) (Fig. 4I and J; Fig. S2G and H). These results suggest that the decrease in viral RNA levels upon overexpression of *miR-216b-5p* and *miR-1-3p* is due to their direct binding to 3′ UTR and NS5 RNA fragments of the JEV and WNV genome. Moreover, RIP analysis indicated that overexpression of *miR-216b-5p/miR-1-3p* in WNV*-*infected SH-SY5Y resulted in enhanced binding of Ago2 to WNV RNA in comparison to WNV-infected SH-SY5Y cells transfected with miR-mimic-NS (Fig. 4K and L). These results suggest that *miR-216b-5p* and *miR-1-3p* binding to viral RNA fragments results in enhanced binding of the viral genome to Ago2 and its possible degradation by RNA-induced silencing complex (RISC).

### *miR-216b-5p* and *miR-1-3p* overexpression attenuates JEV or WNV-infection-induced neuronal cell death

**Fig 5:**
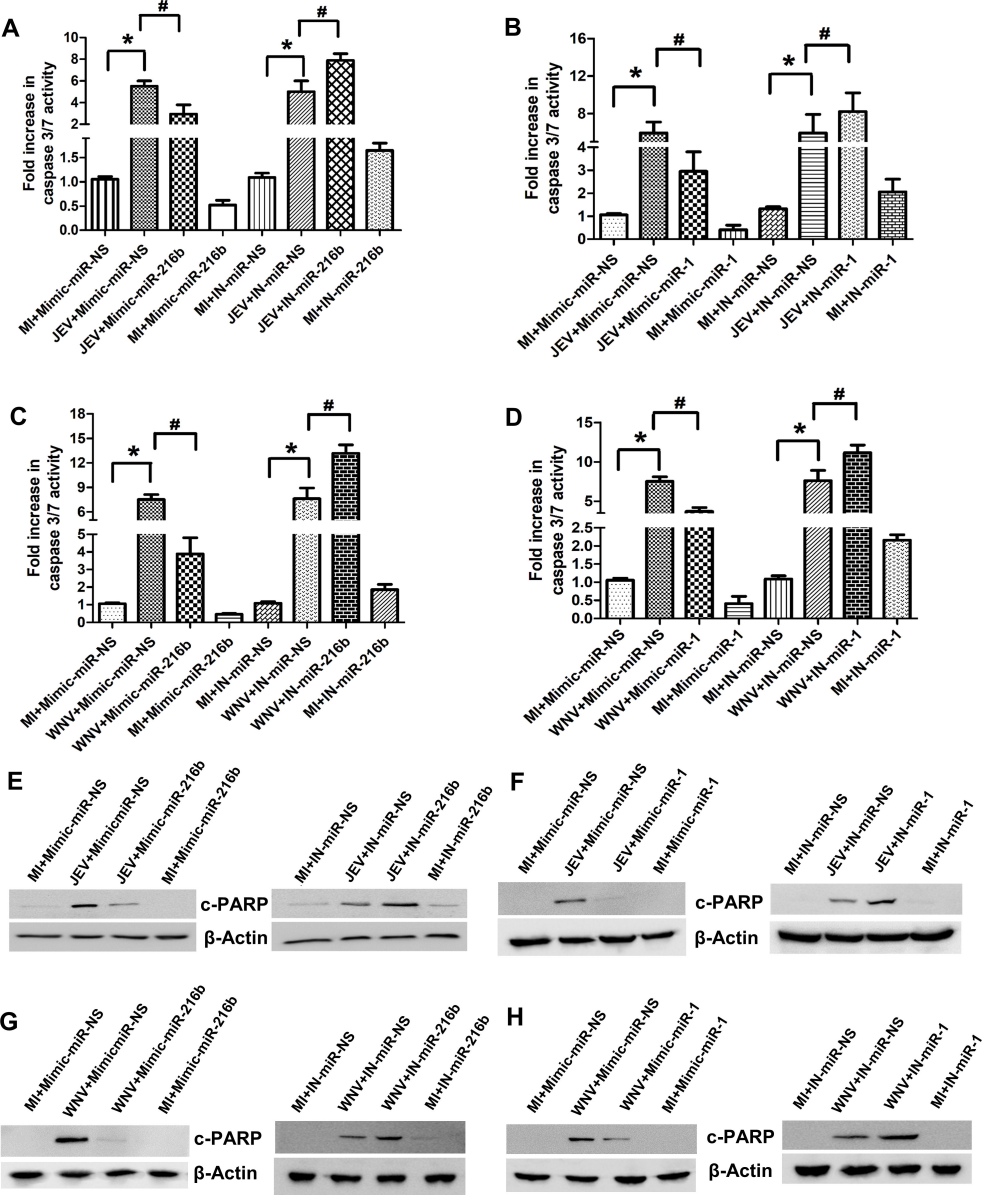
*miR-216b-5p* and *miR-1-3p* overexpression attenuates JEV/WNV-induced cell death. (**A**) *miR-216b-5p* overexpression protects SH-SY5Y cells from JEV-induced apoptosis. SH-SY5Y cells were transfected with either mimic/inhibitor of *miR-216b-5p* or mimic-miR-NS/IN-miR-NS, as indicated, followed by MI or JEV infection. Caspase-3/7 activity was determined at 60 hpi. (**B**) *miR-1-3p* overexpression protects SH-SY5Y cells from JEV-induced apoptosis. SH-SY5Y cells were transfected with either mimic/inhibitor of *miR-1-3p* or mimic-miR-NS/IN-miR-NS, as indicated, followed by MI or JEV infection. Caspase-3/7 activity was determined at 60 hpi. (**C**) *miR-216b-5p* overexpression protects SH-SY5Y cells from WNV-induced apoptosis. SH-SY5Y cells were transfected with mimic/inhibitor of *miR-216b-5p* or mimic-miR-NS/IN-miR-NS, as indicated, followed by MI or WNV infection. Caspase-3/7 activity was determined at 60 hpi. (**D**) *miR-1-3p* overexpression protects SH-SY5Y cells from WNV-induced apoptosis. SH-SY5Y cells were transfected with mimic/inhibitor of *miR-1-3p* or mimic-miR-NS/IN-miR-NS, as indicated, followed by MI or WNV infection. Caspase-3/7 activity was determined at 60 hpi. (**E**) *miR-216b-5p* overexpression reduces JEV-induced cleaved PARP levels in SH-SY5Y cells. SH-SY5Y cells were transfected with mimic/inhibitor of *miR-216b-5p* or mimic-miR-NS/IN-miR-NS, as indicated, followed by MI or JEV infection. (**F**) *miR-1-3p* overexpression reduces JEV-induced cleaved PARP levels in SH-SY5Y cells. SH-SY5Y cells were transfected with mimic/inhibitor of *miR-1-3p* or mimic-miR-NS/IN-miR-NS, as indicated, followed by MI or JEV infection. (**G**) *miR-216b-5p* overexpression reduces WNV-induced cleaved PARP levels in SH-SY5Y cells. SH-SY5Y cells were transfected with either mimic/inhibitor of *miR-216b-5p* or mimic-miR-NS/IN-miR-NS, as indicated, followed by MI or WNV infection. (**H**) *miR-1-3p* overexpression reduces WNV-induced cleaved PARP levels in SH-SY5Y cells. SH-SY5Y cells were transfected with either mimic/inhibitor of *miR-1-3p* or mimic-miR-NS/IN-miR-NS, as indicated, followed by MI or WNV infection. Data information: error bars represent the mean ± SEM from three independent experiments. Statistical comparisons between groups were made using Student’s *t*-test (For A and **B**) *Significant change compared to respective MI+Mimic-miR-NS/MI+IN-miR-NS; ^#^significant change compared to respective JEV+Mimic-miR-NS/JEV+IN-miR-NS. (For C and **D**) *Significant change compared to the corresponding MI+Mimic-miR-NS/IN-miR-NS; ^#^significant change compared to the corresponding WNV+mimic-miR-NS/WNV+IN-miR-NS. (For E–H). The relative levels of cleaved PARP were determined by Western blotting at 60 hpi, and a representative blot is shown from three independent experiments with similar results. Blots were reprobed for β-actin to establish equal loading.

Because *miR-216b-5p* and *miR-1-3p* inhibit JEV and WNV replication (Fig. 3), we evaluated the impact of their overexpression on virus-induced neuronal cell death by measuring caspase 3/7 activity and cleaved-PARP protein expression. *miR-216b-5p* or *miR-1-3p* overexpression in SH-SY5Y cells significantly attenuated JEV or WNV-induced caspase 3/7 activity (Fig. 5A through D). Moreover, the depletion of *miR-216b-5p* or *miR-1-3p* enhanced JEV or WNV-induced caspase 3/7 activity in SH-SY5Y cells (Fig. 5A through D). In agreement with these findings, overexpression of either *miR-216b-5p* or *miR-1-3p* in JEV/WNV-infected SH-SY5Y cells caused a significant reduction in cleaved PARP protein levels compared to JEV/WNV-infected SH-SY5Y cells transfected with mimic-miR-NS (Fig. 5E through H; Fig. S3A, C, E, and G). In line with this, the inhibition of *miR-216b-5p* or *miR-1-3p* in SH-SY5Y cells enhances virus-induced cleaved PARP protein expression (Fig. 5E through H; Fig. S3B, D, F, and H). These results suggest that *miR-216b-5p* or *miR-1-3p* overexpression attenuates virus-induced neuronal cell death.

### *JINR1* mediates *miR-216b-5p* and *miR-1-3p* downregulation during JEV and WNV infection

**Fig 6:**
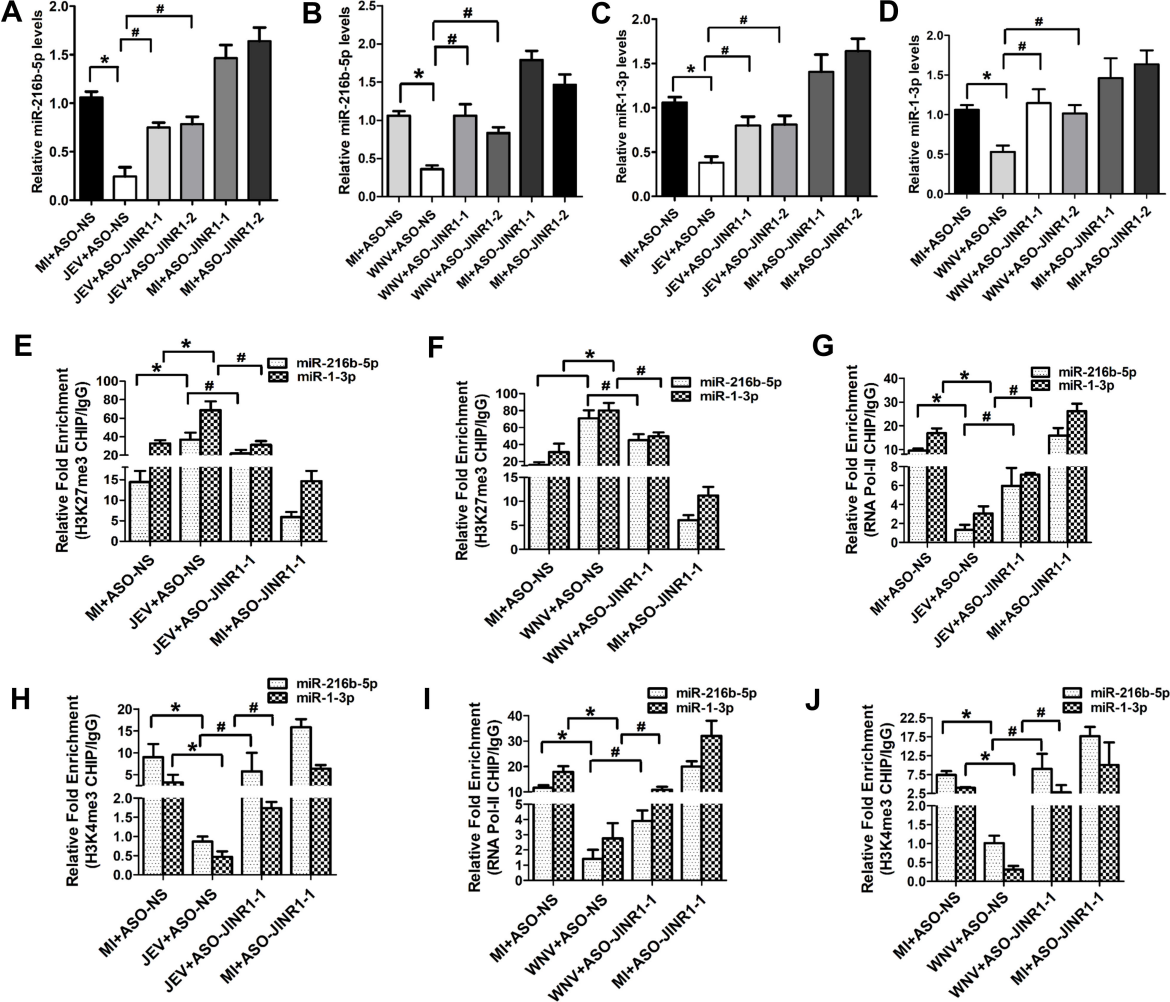
*JINR1* is involved in the transcription repression of *miR-216b-5p* and *miR-1-3p* during JEV/WNV infection. (**A**) *JINR1* inhibits the expression of the mature form of *miR-216b-5p* upon JEV infection. (**B**) *JINR1* inhibits the expression of the mature form of *miR-216b-5p* upon WNV infection. (**C**) *JINR1* inhibits the expression of the mature form of *miR-1-3p* upon JEV infection. (**D**) *JINR1* inhibits the expression of the mature form of *miR-1-3p* upon WNV infection. (**E**) *JINR1* increases H3K27me3 recruitment at the promoter of *miR-216b-5p*/*miR-1-3p* during JEV infection. Relative enrichment of H3K27me3 at the *miR-216-5p*/*miR-1-3p* promoter in MI or JEV-infected SH-SY5Y cells transfected with ASO-*JINR1* or ASO-NS determined by ChIP-qRT-PCR at 48 hpi. (**F**) *JINR1* increases H3K27me3 recruitment at the promoter of *miR-216b-5p*/*miR-1-3p* during WNV infection. Relative enrichment of H3K27me3 at the *miR-216-5p*/*miR-1-3p* promoter in MI or WNV-infected SH-SY5Y cells transfected with ASO-*JINR1* or ASO-NS determined by ChIP-qRT-PCR at 48 hpi. (**G**) *JINR1* reduces the RNA Pol II binding at the promoter of *miR-216b-5p*/*miR-1-3p* during JEV infection. Relative enrichment of RNA Pol II at the *miR-216-5p*/*miR-1-3p* promoter in MI or JEV-infected SH-SY5Y cells transfected with ASO-*JINR1* or ASO-NS determined by ChIP-qRT-PCR at 36 hpi. (**H**) *JINR1* reduces H3K4me3 recruitment at the promoter of *miR-216-5p*/*miR-1-3p* during JEV infection. Relative enrichment of H3K4me3 at the *miR-216-5p*/*miR-1-3p* promoter in MI or JEV-infected SH-SY5Y cells transfected with ASO-*JINR1* or ASO-NS determined by ChIP-qRT-PCR at 36 hpi. (**I**) *JINR1* reduces the RNA Pol II binding at the promoter of *miR-216b-5p*/*miR-1-3p* during WNV infection. Relative enrichment of RNA Pol II at the *miR-216-5p*/*miR-1-3p* promoter in MI or WNV-infected SH-SY5Y cells transfected with ASO-*JINR1* or ASO-NS determined by ChIP-qRT-PCR at 36 hpi. (**J**) *JINR1* reduces H3K4me3 recruitment at the promoter of *miR-216-5p*/*miR-1-3p* during WNV infection. Relative enrichment of H3K4me3 at the *miR-216-5p*/*miR-1-3p* promoter in MI or WNV-infected SH-SY5Y cells transfected with ASO-*JINR1* or ASO-NS determined by ChIP-qRT-PCR at 36 hpi. Data information: error bars represent the mean ± SEM from three independent experiments. Statistical comparisons between groups were made using the Student’s *t*-test. (For A–D) qRT-PCR analysis of indicated transcript upon *JINR1* knockdown in SH-SY5Y cells at 48 hpi. (For E–J) DNA samples were analyzed by qRT-PCR. Enrichment values are relative to MI+ASO-NS-IgG IP. (For A, **C, E, G, and H**) *Significant change compared to respective MI+ASO-NS; ^#^significant change compared to respective JEV+ASO-NS. (For B, **D, F, I, and J**) *Significant change compared to the corresponding MI+ASO-NS; ^#^significant change compared to the corresponding WNV+ASO-NS.

Because *JINR1* regulates transcription during JEV, WNV, and DENV infection (38), and because JEV and WNV infection results in the inhibition of transcription of *miR-216b-5p* and *miR-1-3p*, we analyzed the levels of primary, precursor, and mature transcripts of *miR-216b-5p* and *miR-1-3p* upon *JINR1* depletion in JEV- or WNV-infected SH-SY5Y cells. *JINR1* knockdown significantly rescued the JEV- or WNV-mediated decrease in the expression of primary, precursor, and mature *miR-216b-5p* and *miR-1-3p* transcripts (Fig. 6A through D; Fig. S4). In agreement with this, overexpression of *JINR1* during JEV or WNV infection in SH-SY5Y cells significantly reduces the primary, precursor, and mature *miR-216b-5p* and *miR-1-3p* transcript levels compared to the cells transfected with a control vector (Con-Vec) (Fig. S5). These results suggested that *JINR1* is involved in JEV- or WNV-mediated transcription inhibition of *miR-216b-5p* and *miR-1-3p*. To further confirm that *JINR1* is engaged in the JEV- or WNV-mediated transcription repression of *miR-216b-5p* and *miR-1-3p*, we performed ChIP qRT-PCR to assess the binding of RNA-Pol-II, H3K4me3, and H3K27me3 at the promoters of *miR-216b-5p* and *miR-1-3p* upon *JINR1* depletion. *JINR1* silencing attenuates the virus-mediated increase in H3K27me3 binding at the promoters of *miR-216b-5p* and *miR-1-3p* (Fig. 6E and F). Moreover, *JINR1* knockdown prevented the JEV- or WNV-mediated reduction in RNA-Pol-II and H3K4me3 binding at the promoters of *miR-216b-5p* and *miR-1-3p* (Fig. 6G through J). Additionally, *JINR1* silencing had no significant change in the expression of primary, precursor, and mature *miR-370-5p* and *miR-30e-3p* transcripts (Fig. S6 and S7). Neither did *JINR1* silencing result in any change in the H3K27me3, RNA-Pol-II, and H3K4me3 binding at the promoters of *miR-370-5p* and *miR-30e-3p* (Fig. S8 and S9). These results suggest that *JINR1* specifically mediates transcription repression of *miR-216b-5p* and *miR-1-3p* during JEV and WNV infection.

### RBM10 regulates *miR-216b-5p* and *miR-1-3p* transcription inhibition during JEV/WNV infection

**Fig 7:**
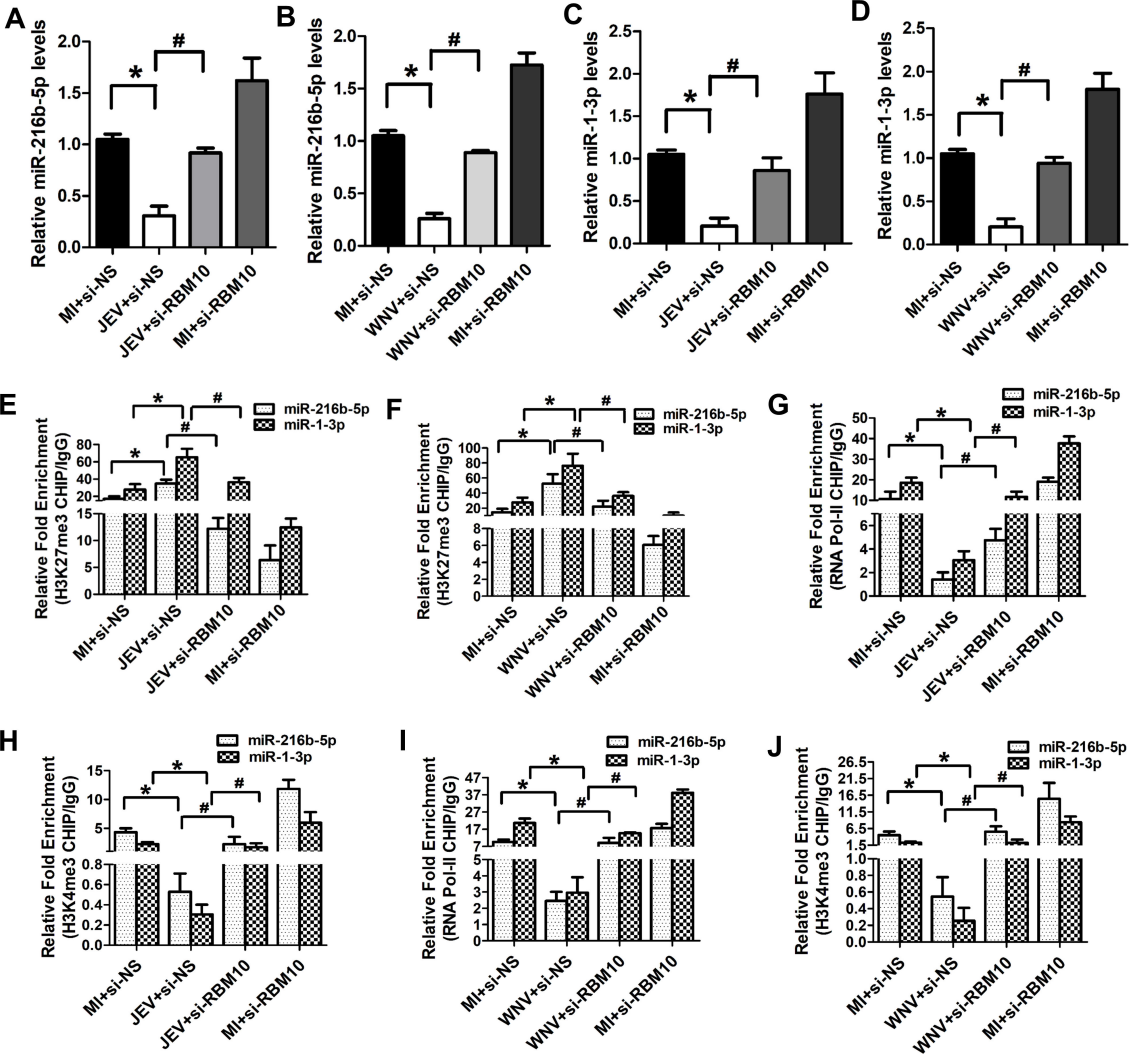
RBM10 is involved in the transcription repression of *miR-216b-5p* and *miR-1-3p* during JEV/WNV infection. (**A**) RBM10 inhibits the expression of the mature form of *miR-216b-5p* upon JEV infection. (**B**) RBM10 inhibits the expression of the mature form of *miR-216b-5p* upon WNV infection. (**C**) RBM10 inhibits the expression of the mature form of *miR-1-3p* upon JEV infection. (**D)** RBM10 inhibits the expression of the mature form of *miR-1-3p* upon WNV infection. (**E**) RBM10 increases H3K27me3 recruitment at the promoter of *miR-216b-5p/miR-1-3p* during JEV infection. Relative enrichment of H3K27me3 at the *miR-216-5p/miR-1-3p* promoter in MI or JEV-infected SH-SY5Y cells transfected with si-RBM10 or si-NS determined by ChIP-qRT-PCR at 36 hpi. (**F**) RBM10 increases H3K27me3 recruitment at the promoter of *miR-216b-5p/miR-1-3p* during WNV infection. Relative enrichment of H3K27me3 at the *miR-216-5p/miR-1-3p* promoter in MI or WNV-infected SH-SY5Y cells transfected with si-RBM10 or si-NS determined by ChIP-qRT-PCR at 36 hpi. (**G**) RBM10 reduces the RNA Pol II binding at the promoter of *miR-216b-5p/miR-1-3p* during JEV infection. Relative enrichment of RNA Pol II at the *miR-216-5p/miR-1-3p* promoter in MI or JEV-infected SH-SY5Y cells transfected with si-RBM10 or si-NS determined by ChIP-qRT-PCR at 36 hpi. (**H**) RBM10 reduces H3K4me3 recruitment at the promoter of *miR-216-5p/miR-1-3p* during JEV infection. Relative enrichment of H3K4me3 at the *miR-216-5p/miR-1-3p* promoter in MI or JEV-infected SH-SY5Y cells transfected with si-RBM10 or si-NS determined by ChIP-qRT-PCR at 36 hpi. (**I**) RBM10 reduces the RNA Pol II binding at the promoter of *miR-216b-5p/miR-1-3p* during WNV infection. Relative enrichment of RNA Pol II at the *miR-216-5p/miR-1-3p* promoter in MI or WNV-infected SH-SY5Y cells transfected with si-RBM10 or si-NS determined by ChIP-qRT-PCR 36 hpi. (**J**) RBM10 reduces H3K4me3 recruitment at the promoter of *miR-216-5p/miR-1-3p* during WNV infection. Relative enrichment of H3K4me3 at the *miR-216-5p/miR-1-3p* promoter in MI or WNV-infected SH-SY5Y cells transfected with si-RBM10 or si-NS determined by ChIP-qRT-PCR at 36 hpi. Data information: error bars represent the mean ± SEM from three independent experiments. Statistical comparisons between groups were made using the Student’s *t*-test. (For A–D) qRT-PCR analysis of the indicated transcript upon RBM10 knockdown in SH-SY5Y cells at 48 hpi. (For E–J) DNA samples were analyzed by qRT-PCR. Enrichment values are relative to MI+si-NS-IgG IP. (For A, **C, E, G, and H**) *Significant change compared to respective MI+si-NS; ^#^significant change compared to respective JEV+si-NS. (For B, **D, F, I, and J**) *Significant change compared to the corresponding MI+si-NS; ^#^significant change compared to the corresponding WNV+si-NS.

Because we had previously shown that RBM10 interacts with *JINR1* and promotes its expression in SH-SY5Y cells during JEV/WNV infection (38), we asked if RBM10 also regulates *miR-216b-5p* and *miR-1-3p* transcription inhibition during JEV/WNV infection. As with *JINR1* depletion, RBM10 silencing also prevented JEV and WNV infection-mediated decrease in the expression of primary, precursor, and mature transcripts of *miR-216b-5p* and *miR-1-3p* (Fig. 7A through D; Fig. S10). RBM10 overexpression analysis in SH-SY5Y cells during JEV/WNV infection also confirmed that RBM10 is involved in inhibiting the expression of *miR-216b-5p* and *miR-1-3p* (Fig. S11). RBM10 knockdown in JEV- or WNV-infected cells significantly reduces H3K27me3 binding at the *miR-216b-5p* and *miR-1-3p* promoters compared to control cells (Fig. 7E and F). Furthermore, RBM10 depletion in JEV- or WNV-infected cells enhances RNA-Pol-II and H3K4me3 binding at the promoters of *miR-216b-5p* and *miR-1-3p* in comparison to JEV/WNV-infected cells transfected with si-NS (Fig. 7G through J). RBM10 silencing did not result in any noticeable change in the expression of primary, precursor, and mature *miR-370-5p* and *miR-30e-3p* transcripts during viral infection (Fig. S12 and S13). In line with these findings, we also did not observe any change in the binding of H3K27me3, RNA-Pol-II, and H3K4me3 at their promoters with or without infection (Fig. S14 and S15). These results confirm that along with *JINR1,* RBM10 is also involved in the transcription inhibition of *miR-216b-5p* and *miR-1-3p*. Because RBM10 not only interacts with *JINR1* but also regulates its expression during viral infection (38), it remains unclear whether transcriptional inhibition of *miR-216b-5p* and *miR-1-3p* by RBM10 is due to its effect on *JINR1* expression or its interaction with it.

### *miR-216b-5p* and *miR-1-3p* regulate *JINR1* levels in SH-SY5Y cells

*JINR1* is known to interact with *miR-216b-5p* and *miR-1-3p* (39, 40); hence, we performed dual luciferase assays to verify the binding of *miR-216b-5p/miR-1-3p* to *JINR1*. To this end, we generated full-length wild-type *JINR1* (pmirGLO-WT-JINR1) reporter and mutant *JINR1* constructs with substitution in miR-216/miR-1 binding sites (pmirGLO-mut-miR-1-JINR1/pmirGLO-mut-miR-216-JINR1) and performed the luciferase assays in HEK293T. *miR-216b-5p* overexpressing cells had ~30% less pmirGLO-WT-JINR1 reporter activity than cells overexpressing mimic-miR-NS (Fig. S16A and B). The luciferase activity of cells co-transfected with pmirGLO-mut-miR-216-JINR1 luciferase reporter construct and *miR-216b-5p* mimic was comparable to cells co-transfected with mimic-miR-NS and pmirGLO-WT-JINR1 luciferase reporter construct (Fig. S16A and B). *miR-1-3p* overexpression resulted in ~39% less pmirGLO-WT-JINR1 reporter activity than mimic-miR-NS overexpression (Fig. S16C and D). The decrease in luciferase reporter activity was abolished upon mutation of the *miR-1-3p* binding site in the *JINR1* (Fig. S16C and D). In addition, RIP analysis revealed that JEV/WNV-infected cells overexpressing *miR-1-3p*/*miR-216b-5p* had significantly higher binding of *JINR1* to Ago2 than JEV/WNV-infected cells overexpressing mimic-miR-NS. (Fig. S16E through H). The binding of Ago2 to *JINR1* in cells overexpressing mimic-miR-NS during JEV/WNV infection was considerably less than that of mock-infected cells transfected with mimic-miR-NS (Fig. S16E through H). This is likely due to the downregulation of *miR-1-3p*/*miR-216b-5p* during viral infection.

Next, we evaluated the impact of *miR-216b-5p* and *miR-1-3p* overexpression and silencing on *JINR1* expression in SH-SY5Y cells. Transfection of *miR-216b-5p/miR-1-3p* mimics in SH-SY5Y cells significantly reduced *JINR1* expression compared to cells transfected with mimic-miR-NS (Fig. S16I and J). In agreement with this, cells transfected with *miR-216b-5p* or *miR-1-3p* inhibitors had significantly higher *JINR1* expression compared to cells transfected with IN-miR-NS (Fig. S16I and J).

### *JINR1* modulates JEV/WNV replication in neuronal cells via the *miR-1-3p*/DDX5 axis

**Fig 8:**
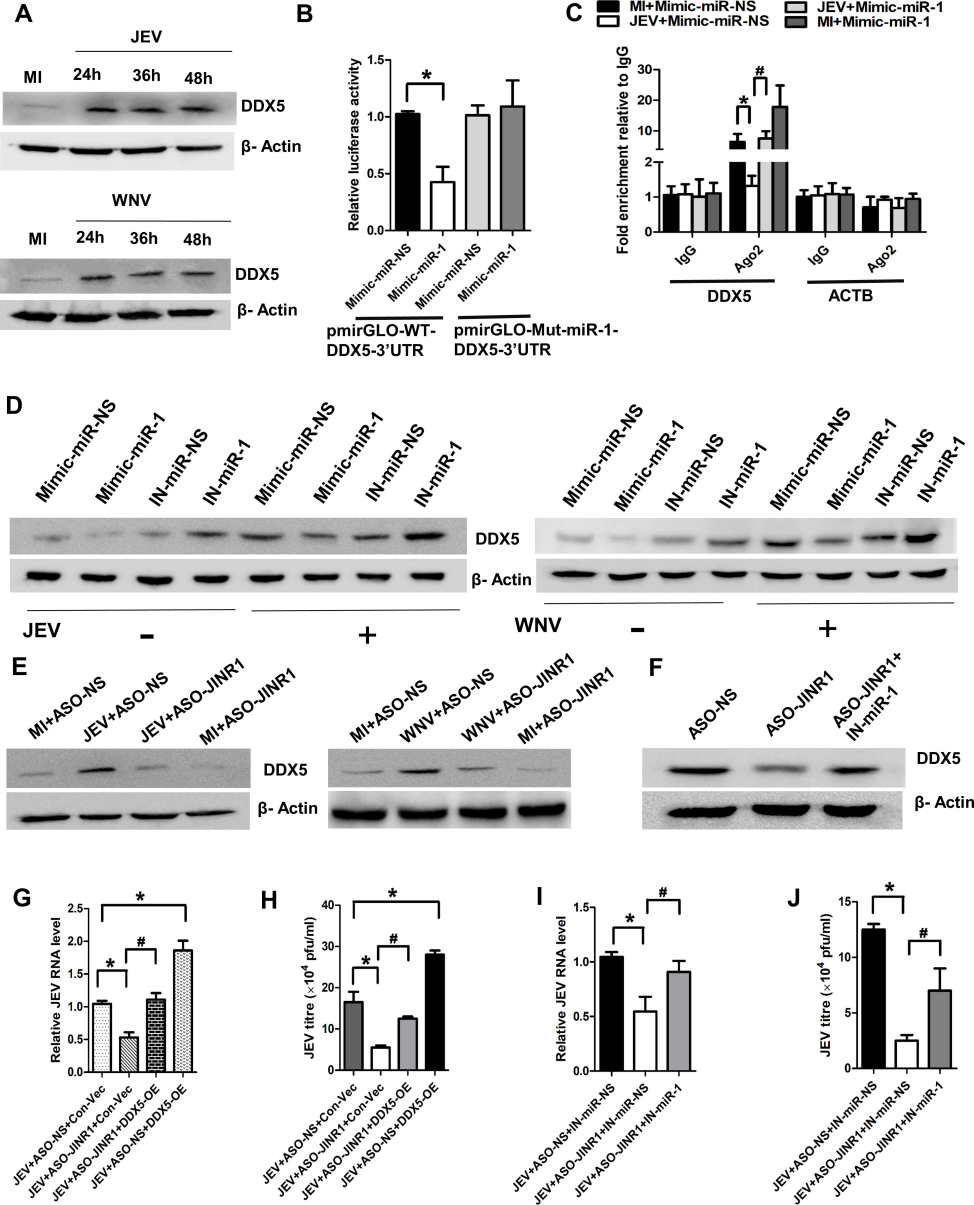
*JINR1* promotes DDX5 expression during JEV and WNV infection by acting as ceRNA for *miR-1-3p*. (**A**) JEV/WNV infection increases DDX5 levels in SH-SY5Y cells. SH-SY5Y cells were infected with JEV/WNV (5 MOI) at indicated time points. (**B**) *miR-1-3p* binds with DDX5-3′ UTR. Dual luciferase activity was measured in HEK-293T cells co-transfected with pmiRGLO-WT-DDX5-3′ UTR or pmiRGLO-mut-miR-1-DDX5-3′ UTR construct and *miR-1-3p* mimic/mimic-miR-NS. Luminescence signals were measured at 36 hours post-transfection. (**C**) *miR-1-3p* overexpression promotes the binding of DDX5 RNA with Ago2 during JEV infection in SH-SY5Y cells. Anti-Ago2 RIP was performed in MI and JEV-infected SH-SY5Y cells, transiently overexpressing *miR-1-3p* or mimic-NS, followed by qRT-PCR to detect DDX5 and ACTB associated with Ago2. The data shown are normalized to input and represented as fold enrichment relative to MI+Mimic-miR-NS-IgG IP/JEV+Mimic-miR-NS-IgG IP. Values represent the mean ± SEM from three independent experiments. (**D**) *miR-1-3p* overexpression reduces JEV/WNV-induced DDX5 levels in SH-SY5Y cells. SH-SY5Y cells were transfected with either mimic/inhibitor of *miR-1-3p* or mimic-miR-NS/IN-miR-NS, as indicated, followed by MI or JEV/WNV infection. (**E**) *JINR1* promotes DDX5 levels during JEV/WNV infection in SH-SY5Y cells. SH-SY5Y cells were transfected with either ASO-NS or ASO-*JINR1,* as indicated, followed by MI or JEV/WNV infection. (**F**) Rescue of DDX5 levels due to *JINR1* depletion in SH-SY5Y cells upon *miR-1-3p* inhibition. SH-SY5Y cells were co-transfected with either ASO-NS and IN-miR-NS, IN-miR-NS and ASO-*JINR1,* or ASO-*JINR1* and IN-*miR-1-3p*. (**G**) DDX5 overexpression prevents the reduction in JEV RNA levels due to *JINR1* depletion in SH-SY5Y cells. SH-SY5Y cells were co-transfected with either ASO-NS and Con-Vec or ASO-*JINR1* and Con-Vec or ASO-*JINR1* and DDX5-OE or ASO-NS and DDX5-OE, and infected with JEV. Viral replication was determined by quantifying the intracellular levels of JEV RNA at 48 hpi. (**H**) DDX5 overexpression prevents the reduction in JEV titer due to *JINR1* depletion in SH-SY5Y cells. Quantification of viral titer from SH-SY5Y cells co-transfected with either ASO-NS and Con-Vec or ASO-*JINR1* and Con-Vec or ASO-*JINR1* and DDX5-OE or ASO-NS and DDX5-OE, and infected with JEV. (**I**) *miR-1-3p* silencing prevents the reduction in JEV RNA levels due to *JINR1* depletion in SH-SY5Y cells. SH-SY5Y cells were co-transfected with either ASO-NS and IN-miR-NS, IN-miR-NS and ASO-*JINR1,* or ASO-*JINR1* and *miR-1-3p* inhibitor, and infected with JEV. Viral replication was determined by quantifying the intracellular levels of JEV RNA at 48 hpi. RNA samples were analyzed by qRT–PCR. (**J**) *miR-1-3p* inhibition prevents the reduction in JEV titer due to *JINR1* depletion in SH-SY5Y cells. Quantification of viral titer from SH-SY5Y cells co-transfected with either ASO-NS+IN-miR-NS, IN-miR-NS+ASO-*JINR1,* or ASO-*JINR1+miR-1-3p* inhibitor, and infected with JEV. Data information: error bars represent the mean ± SEM from three independent experiments. Statistical comparisons between groups were made using the Student’s *t*-test. (For B) *Significant change compared to Mimic-miR-NS; ^#^significant change compared to IN-miR-NS. (For B) *Significant change compared to Mimic-miR-NS. (For C) *Significant change compared to MI+Mimic-miR-NS-IgG-IP; ^#^significant change compared to JEV+Mimic-miR-NS-IgG-IP. (For A, **D–F**) The relative levels of DDX5 were determined by Western blotting at 48 hpi, and a representative blot is shown from three independent experiments with similar results. Blots were reprobed for β-actin to establish equal loading. (For G and H) *Significant change compared to respective JEV+ASO-NS+Con-Vec; ^#^significant change compared to respective JEV+ASO-*JINR1*+Con-Vec. (For I and J) *Significant change compared to the corresponding JEV+ASO-NS+IN-miR-NS; ^#^significant change compared to the corresponding JEV+ASO-*JINR1*+IN-miR-NS.

Besides directly binding to the virus genome, miRNAs can also bind to host transcripts to influence viral replication and pathogenesis (50). Because *JINR1* binds to *miR-1-3p* and regulates JEV/WNV replication, we searched for *miR-1-3p* host target genes that regulate viral replication (47). Among the several *miR-1-3p* target genes identified using the miRWalk database, we found DDX5 is one of the genes that regulate JEV replication. DDX5 positively regulates JEV replication in BHK-21 cells by binding to the JEV 3′ UTR region (53). IntaRNA analysis revealed five binding sites between DDX5 3′ UTR and *miR-1-3p* sequences (Fig. S17). To establish the involvement of DDX5 during JEV/WNV infection in SH-SY5Y cells, we first did a time course analysis of its mRNA and protein expression during viral infection. DDX5 transcript and protein expression increased in a time-dependent manner during JEV and WNV infection (Fig. 8A; Fig. S18A through D). Next, we confirmed if DDX5 promotes JEV and WNV replication in SH-SY5Y cells. Consistent with the pro-viral function of DDX5, we observed that overexpression of DDX5 increases both JEV and WNV intracellular RNA levels in SH-SY5Y cells in comparison to JEV/WNV-infected cells transfected with Con-Vec (Fig. S18E and F). To confirm the regulation of DDX5 by *miR-1-3p* in SH-SY5Y cells, we measured the expression of endogenous DDX5 mRNA levels in SH-SY5Y cells transfected with *miR-1-3p* mimics or inhibitors. Overexpression of *miR-1-3p* significantly reduced DDX5 (~64%) mRNA levels compared to cells transfected with mimic-miR-NS (Fig. S18G). In contrast, the transfection of *miR-1-3p* inhibitor in SH-SY5Y cells enhanced the mRNA expression of DDX5 (~1.91-fold) compared to cells transfected with IN-miR-NS (Fig. S18G). Next, we performed the dual luciferase and UV Ago2 RIP assays to verify the binding of *miR-1-3p* with DDX5-3′ UTR. Cells co-transfected with *miR-1-3p* mimic and DDX5 3′ UTR (pmirGLO-WT-DDX5-3′ UTR) luciferase reporter construct had ~58% less reporter activity than cells co-transfected with mimic-miR-NS and pmirGLO-WT-DDX5-3′ UTR reporter construct (Fig. 8B). Cells co-transfected with mutant DDX5-3′ UTR (pmirGLO-mut-miR-1-DDX5-3′ UTR) luciferase reporter construct and *miR-1-3p* mimic had no significant change in luciferase activity compared to cells co-transfected with mimic-miR-NS and pmirGLO-WT-DDX5-3′ UTR luciferase reporter construct (Fig. 8B).

UV Ago2 RIP assay shows that the binding of Ago2 to DDX5 in cells overexpressing mimic-miR-NS during JEV/WNV infection was considerably less than that of mock-infected cells transfected with mimic-miR-NS; this is likely due to the downregulation of *miR-1-3p* during viral infection (Fig. 8C; Fig. S18H). As expected, the Ago2 binding to DDX5 was significantly enhanced in SH-SY5Y cells overexpressing *miR-1-3p* than SH-SY5Y cells overexpressing *miR-*mimic-NS, both with and without viral infection (Fig. 8C; Fig. S18H). These results imply that *miR-1-3p* directly binds to DDX5-3′ UTR to regulate its expression negatively.

Because JEV and WNV infection results in the downregulation of *miR-1-3p* expression and because *miR-1-3p* targets DDX5, we evaluated the impact of *miR-1-3p* overexpression and silencing on DDX5 protein levels during JEV/WNV infection. As expected, *miR-1-3p* overexpression significantly reduced the basal and JEV/WNV-induced DDX5 protein expression in SH-SY5Y cells (Fig. 8D; Fig. S19A and C). *miR-1-3p* inhibition enhanced the basal and JEV/WNV-induced DDX5 protein expression (Fig. 8D; Fig. S19B and D). Because *JINR1* sponges *miR-1-3p* (Fig. S8B), we asked whether *JINR1* regulates the expression of DDX5 during JEV/WNV infection. Silencing of *JINR1* reduces JEV/WNV-induced expression of DDX5 transcript and protein (Fig. 8E; Fig. S19E through H).

Next, we checked if *miR-1-3p* inhibition can rescue the *JINR1* silencing-mediated downregulation of DDX5 protein levels in SH-SY5Y cells. We observed that *miR-1-3p* inhibition significantly restores DDX5 downregulation caused by *JINR1* depletion (Fig. 8F; Fig. S19I).

Next, we asked if DDX5 overexpression can rescue *JINR1* knockdown-mediated decrease in viral replication. To test this, we evaluated the impact of DDX5 overexpression on JEV/WNV replication in SH-SY5Y cells during *JINR1* depletion. DDX5 overexpression partially rescues the decrease in intracellular JEV and WNV RNA levels due to *JINR1* depletion (Fig. 8G; Fig. S20A). Plaque analysis confirmed that DDX5 overexpression rescues the reduction in infectious viral particle release in the culture supernatant of JEV- and WNV-infected SH-SY5Y cells due to *JINR1* knockdown (Fig. 8H; Fig. S20B).

*miR-1-3p* inhibition partly rescues the reduction in JEV and WNV replication due to *JINR1* silencing, as indicated by intracellular RNA levels and plaque assay in SH-SY5Y cells (Fig. 8I and J; Fig. S21). These results suggest that *JINR1* acts as a ceRNA to sponge *miR-1-3p* for stabilizing DDX5 expression, which promotes JEV and WNV replication in SH-SY5Y cells.

### *JINR1* promotes JEV and WNV replication in neuronal cells via the *miR-216b-5p/GRP78* axis

Next, we searched for target genes of *miR-216b-5p,* which regulate JEV and WNV replication. Interestingly, we identified GRP78 as one of the target genes of *miR-216b-5p* (47). GRP78 facilitates viral entry and promotes the replication of JEV, ZIKV, DENV, and other viruses in their host cells (54–56). Moreover, we have also previously shown that *JINR1* and RBM10-mediated increase in GRP78 expression promotes JEV and WNV replication in SH-SY5Y cells (38).

**Fig 9:**
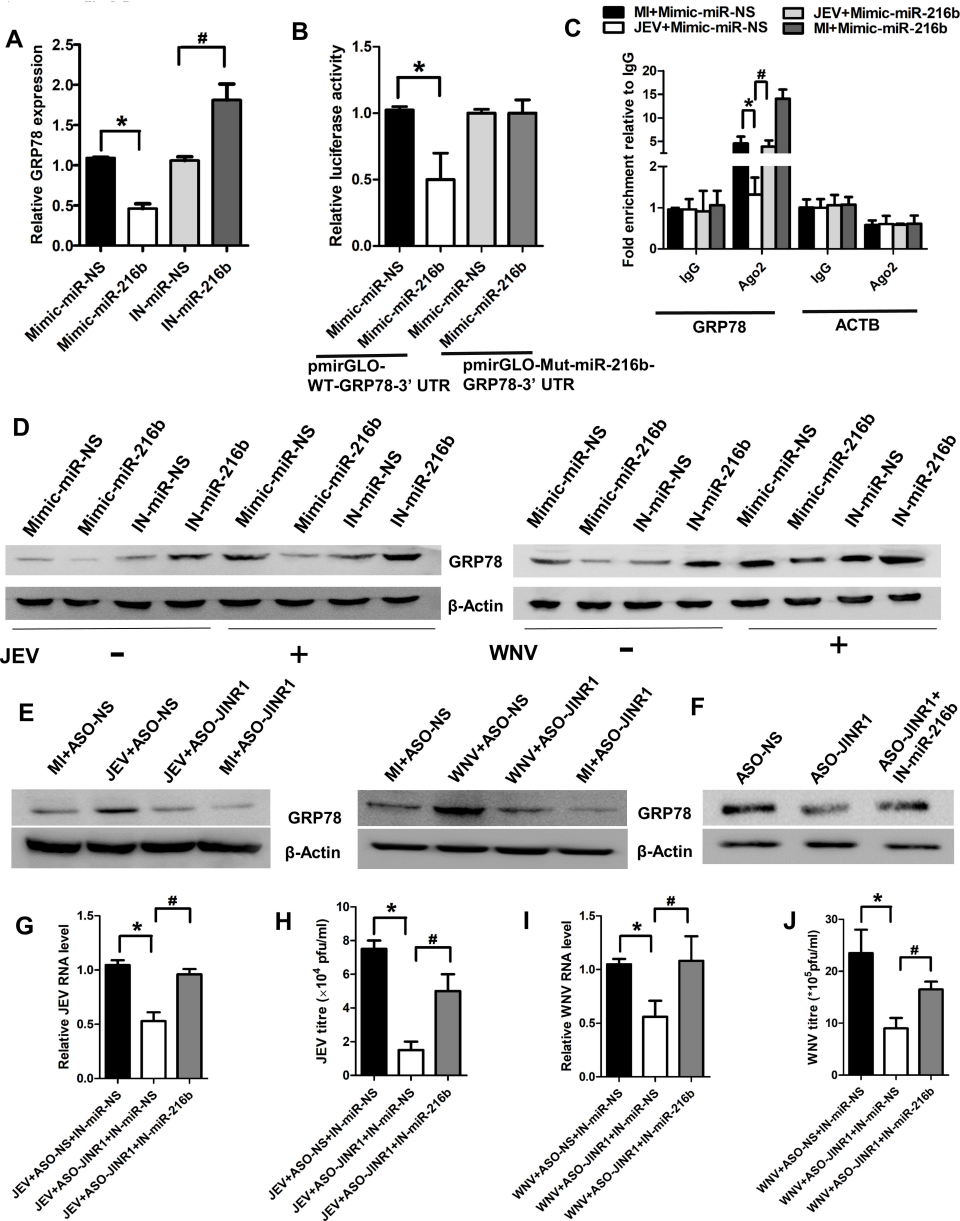
*JINR1* promotes GRP78 expression during JEV and WNV infection by acting as ceRNA for *miR-216b-5p*. (**A**) *miR-216b-5p* negatively regulates GRP78 expression. SH-SY5Y cells were transfected with either mimic/inhibitor of *miR-216b-5p* or mimic-miR-NS/IN-miR-NS, as indicated, and GRP78 transcript levels were measured using qRT-PCR at 48 hpi. (**B**) *miR-216b-5p* binds with GRP78-3′ UTR. Dual luciferase activity was measured in HEK-293T cells co-transfected with pmiRGLO-WT-GRP78-3′ UTR or pmiRGLO-mut-miR-216b-GRP78-3′ UTR construct and *miR-216b-5p* mimic or mimic-miR-NS. Luminescence signals were measured at 36 hours post-transfection. (**C**) *miR-216-5p* overexpression promotes the binding of GRP78 RNA with Ago2 during JEV infection in SH-SY5Y cells. Anti-Ago2 RIP was performed in MI and JEV-infected SH-SY5Y cells, transiently overexpressing *miR-216b-5p* or mimic-NS, followed by qRT-PCR to detect GRP78 and ACTB associated with Ago2. The data shown are normalized to input and represented as fold enrichment relative to MI+Mimic-miR-NS-IgG IP/JEV+Mimic-miR-NS-IgG IP. Values represent the mean ± SEM from three independent experiments. (**D**) *miR-216b-5p* overexpression inhibits GRP78 levels during JEV/WNV infection. SH-SY5Y cells were transfected with either mimic/inhibitor of *miR-216b-5p or* mimic-miR-NS/IN-miR-NS, followed by JEV/WNV infection. (**E**) *JINR1* promotes GRP78 levels during JEV/WNV infection. SH-SY5Y cells were transfected with either ASO-NS or ASO-*JINR1*. (**F**) Rescue of GRP78 protein levels due to *JINR1* depletion in SH-SY5Y cells upon *miR-216b-5p* knockdown. SH-SY5Y cells were co-transfected with either ASO-NS and IN-miR-NS, IN-miR-NS and ASO-*JINR1,* or ASO-*JINR1* and *miR-216b-5p* inhibitor. (**G**) *miR-216b-5p* silencing prevents the reduction in JEV RNA levels due to *JINR1* knockdown in SH-SY5Y cells. SH-SY5Y cells were co-transfected with either ASO-NS and IN-miR-NS, IN-miR-NS and ASO-*JINR1,* or ASO-*JINR1* and *miR-216b-5p* inhibitor, and infected with JEV. Viral replication was determined by quantifying the intracellular levels of JEV RNA at 48 hpi. (**H**) *miR-216b-5p* inhibition prevents the reduction in JEV titer due to *JINR1* knockdown in SH-SY5Y cells. Quantification of viral titer from SH-SY5Y cells co-transfected with either ASO-NS and IN-miR-NS, IN-miR-NS and ASO-*JINR1,* or ASO-*JINR1* and *miR-216b-5p* inhibitor, and infected with JEV. (**I**) *miR-216b-5p* silencing prevents the reduction in WNV RNA levels due to *JINR1* knockdown in SH-SY5Y cells. SH-SY5Y cells were co-transfected with either ASO-NS and IN-miR-NS, IN-miR-NS and ASO-*JINR1,* or ASO-*JINR1* and *miR-216b-5p* inhibitor, and infected with WNV. Viral replication was determined by quantifying the intracellular levels of WNV RNA at 48 hpi. (**J**) *miR-216b-5p* inhibition prevents the reduction in WNV titer due to *JINR1* knockdown in SH-SY5Y cells. Quantification of viral titer from SH-SY5Y cells co-transfected with either ASO-NS and IN-miR-NS, IN-miR-NS and ASO-*JINR1,* or ASO-*JINR1* and *miR-216b-5p* inhibitor, and infected with WNV. Data information: error bars represent the mean ± SEM from three independent experiments. Statistical comparisons between groups were made using the Student’s *t*-test. (For A) *Significant change compared to Mimic-miR-NS; ^#^significant change compared to IN-miR-NS. (For B) *Significant change compared to Mimic-miR-NS. (For C) *Significant change compared to MI+Mimic-miR-NS-IgG-IP; ^#^significant change compared to JEV+Mimic-miR-NS-IgG-IP. (For D–F) The relative levels of GRP78 were determined by Western blotting at 48 hpi, and a representative blot is shown from three independent experiments with similar results. Blots were reprobed for β-actin to establish equal loading. (For G and H) *Significant change compared to respective JEV+ASO-NS+IN-miR-NS; ^#^significant change compared to respective JEV+ASO-*JINR1*+IN-miR-NS. (For I and J) *Significant change compared to the corresponding WNV+ASO-NS+IN-miR-NS; ^#^significant change compared to the corresponding WNV+ASO-*JINR1*+IN-miR-NS.

Using the IntaRNA database, we identified three binding sites between GRP78 3′ UTR and *miR-216b-5p* (Fig. S2). First, we confirmed that GRP78 is a bona fide target of *miR-216b-5p* in SH-SY5Y cells. We measured the GRP78 transcript levels upon *miR-216b-5p* overexpression and downregulation. Overexpression of *miR-216b-5p* significantly reduced GRP78 (~50%) mRNA levels compared to the SH-SY5Y cells transfected with mimic-miR-NS (Fig. 9A). In addition, the transfection of *miR-216b-5p* inhibitor in SH-SY5Y cells significantly enhanced the mRNA expression of GRP78 (~1.67-fold) compared to cells transfected with IN-miR-NS (Fig. 9A). We also confirmed the binding of *miR-216b-5p* to GRP78-3′ UTR using dual luciferase and UV Ago 2 RIP assays. *miR-216b-5p* overexpression results in ~50% less pmirGLO-WT-GRP78-3′ UTR luciferase activity than cells overexpressing mimic-miR-NS (Fig. 9B). *miR-216b-5p* overexpression results in no change in the pmirGLO-mut-miR-216-GRP78-3′ UTR luciferase activity compared to cells overexpressing mimic-miR-NS and pmirGLO-WT-GRP78-3′ UTR reporter construct (Fig. 9B). UV Ago 2 RIP confirmed that binding of GRP78 to Ago2 was significantly enriched upon *miR-216b-5p* overexpression in comparison to *miR-*mimic-NS overexpression in both MI or virus-infected cells (Fig. 9C; Fig. S23A). Collectively, these results confirm that GRP78 is a bona fide *miR-216b-5p* target.

Additionally, *miR-216b-5p* overexpression significantly reduced the basal and JEV/WNV-induced GRP78 protein expression in SH-SY5Y cells compared to their respective controls (Fig. 9D; Fig. S23B and D). *miR-216b-5p* inhibition enhanced the basal and JEV/WNV-induced GRP78 protein levels by approximately twofold compared to their respective controls (Fig. 8C; Fig. S23C and E). Because *JINR1* binds to *miR-216b-5p*, which targets GRP78, we tested the effect of *JINR1* silencing on GRP78 protein levels during viral infection in SH-SY5Y cells. *JINR1* knockdown attenuates the JEV/WNV-mediated increase in GRP78 protein expression compared to SH-SY5Y cells transfected with ASO-NS (Fig. 9E; Fig. S23F and G).

We also tested if *miR-216b-5p* inhibition can rescue the *JINR1* knockdown-mediated downregulation of GRP78 protein levels in SH-SY5Y cells. We observed that the *miR-216b-5p* inhibitor significantly restores GRP78 downregulation caused by *JINR1* depletion (Fig. 9F; Fig. S23H). We have previously shown that GRP78 overexpression rescues *JINR1* knockdown-mediated decrease in JEV and WNV replication (38). Next, we checked the effect of *miR-216b-5p* silencing on JEV and WNV replication during *JINR1* knockdown. Intracellular RNA and plaque assay analysis show that co-transfection of *miR-1-3p* inhibitor with ASO-*JINR1* in SH-SY5Y cells results in significantly higher viral replication in comparison to cells transfected with miR-IN-NS and ASO-*JINR1* (Fig. 9F through I). These results confirm that *JINR1* sponges *miR-216b-5p* to enhance GRP78 expression, which supports viral replication in SH-SY5Y cells.

## DISCUSSION

JEV and WNV often breach the blood-brain barrier and infect neuronal cells, leading to severe outcomes such as encephalitis, neuroinflammation, and death (10, 28, 57–59). JEV and WNV infection results in neuronal apoptosis due to ER stress and the expression of inflammatory cytokines (28, 60–62). Orthoflavivirus infections result in the abnormal expression of lncRNAs and miRNAs in host cells, and they modulate viral infection (29, 38, 63, 64). We have recently shown that JEV/WNV/DENV infection in neuronal cells promotes *JINR1* and RBM10 expression via the NF-κB pathway (38). *JINR1* and RBM10 enhance viral replication and associated neuronal cell death (38). *JINR1* and RBM10 interact with each other and promote self and each other’s expression by regulating the binding of the p65 subunit of NF-κB at their promoters (38). Using the same mechanism, they activate the expression of GRP78 and other genes involved in ER stress and neuroinflammation during JEV infection (38). MicroRNAs *miR-216b-5p* and *miR-1-3p* interact with *JINR1* (39, 40) and regulate influenza infection (65, 66). However, it is unknown if *miR-216b-5p* and *miR-1-3p* and their association with *JINR1* play a role in regulating JEV/WNV infection.

**Fig 10:**
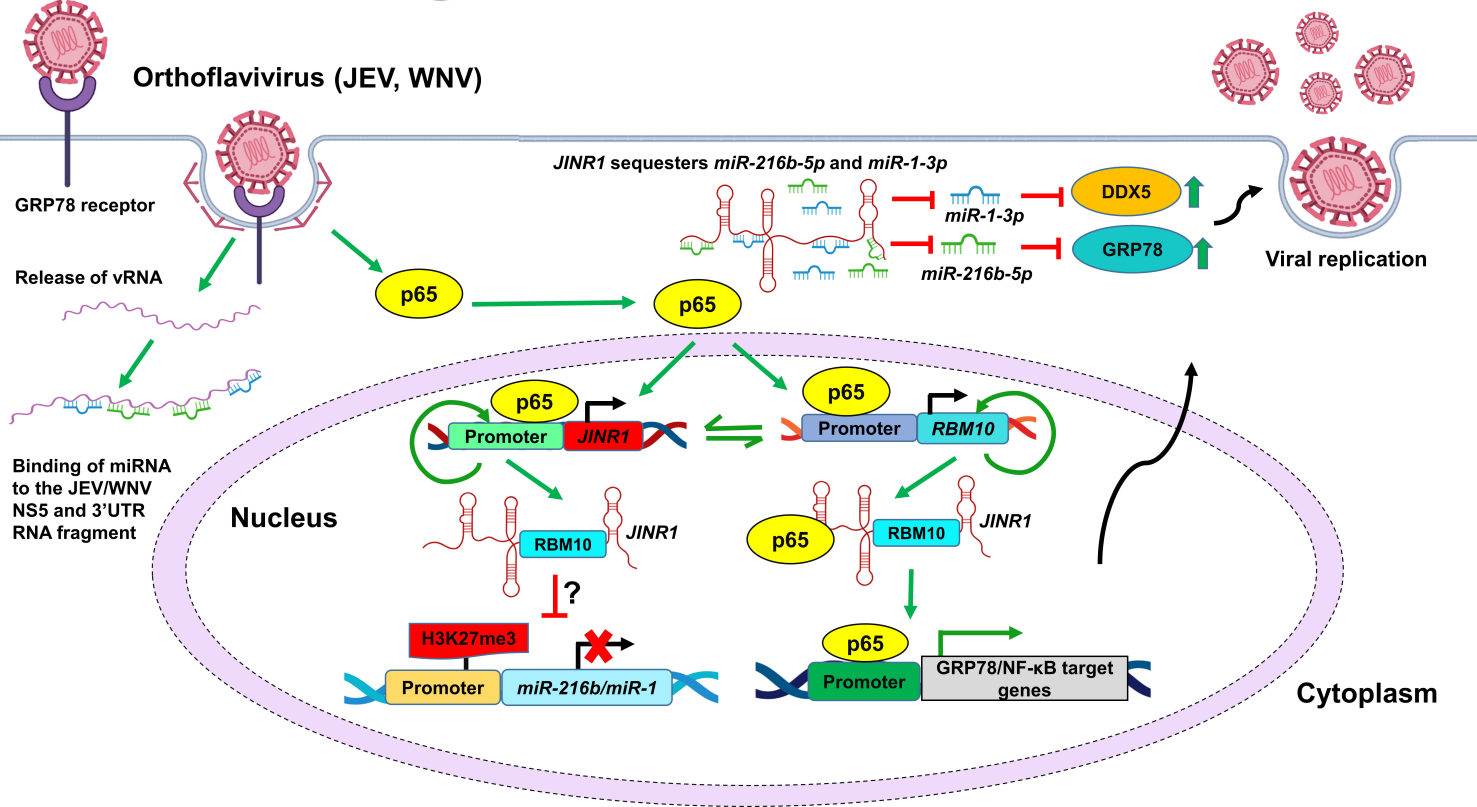
JEV or WNV infection inhibits the expression of *miR-216b-5p* and *miR-1-3p* in SH-SY5Y cells*,* which directly bind with JEV/WNV’s NS5 and 3′ UTR RNA fragments to inhibit viral replication. JEV/WNV-induced lncRNA *JINR1* and its interacting protein, RBM10, are involved in the transcription inhibition of *miR-216b-5p* and *miR-1-3p*. In addition, *JINR1* binds and sequesters *miR-216b-5p* and *miR-1-3p*, resulting in the upregulation of their targets GRP78 and DDX5, respectively, which promote JEV and WNV replication.

Our results show that JEV/WNV infection inhibits the expression of *miR-216b-5p* and *miR-1-3p* in neuronal cells. *miR-216b-5p* and *miR-1-3p* directly bind to the NS5 and 3′ UTR regions of the JEV/WNV genome, and their overexpression during infection reduces viral replication and neuronal cell death (Fig. 10). Interestingly, *miR-1-3p* negatively regulates influenza viral infection by directly binding to the viral genome and the host gene, ATP6V1A (66). However, the impact of the influenza virus on *miR-1-3p* expression is unknown. *miR-216b-5p* also interacts with the influenza virus genome, but its role in regulating influenza replication has not been explored (65). Moreover, overexpression of hepatitis B virus × protein downregulates *miR-216b-5p* expression in liver cells, and *miR-216b-5p* overexpression inhibits hepatitis B virus (HBV) replication (67). These studies confirm that, apart from regulating JEV/WNV infection, *miR-216b-5p* and *miR-1-3p* also modulate infection by other viruses. Interestingly, the expression of *miR-1-3p* is downregulated in neurodegenerative disorders, and their overexpression is beneficial for neuronal cell survival (68–71). This is consistent with our observation of the neuroprotective role of *miR-1-3p* during JEV/WNV infection.

*JINR1* and RBM10 promote transcription of NF-κB target genes during orthoflavivirus infection (38). Here, we show that apart from their role in gene activation, *JINR1* and RBM10 are also involved in the transcription repression of *miR-216b-5p* and *miR-1-3p*. The ability of *JINR1* to both activate and repress gene expression is similar to lncRNA Cox2, which mediates both the activation and repression of distinct classes of immune genes (72). RBM10 is a nuclear RNA-binding protein that regulates pre‐mRNA splicing, mRNA stabilization, and transcription (73–75). RBM10 associates with the histone deacetylase Clr6, a Class I HDAC complex (homolog of mammalian HDAC1 and HDAC2 complex), and chromatin remodelers to promote heterochromatin silencing in fission yeast (76). These findings support our observation of transcription inhibition of *miR-216b-5p* and *miR-1-3p* by RBM10. Even though *JINR1* and RBM10 regulate each other’s expression, considering that they interact with each other (38), we speculate that *JINR1* and RBM10 are part of an RNA-protein complex that regulates miRNA inhibition during JEV/WNV infection. However, further investigations are needed to decipher the exact mechanism of RBM10/*JINR1*-mediated *miR-216b-5p* and *miR-1-3p* transcription repression.

The same lncRNA can regulate gene expression not only at the transcriptional level but also at the post-transcriptional level. For example*,* lncRNA *MALAT1* causes inflammasome activation in microglial cells by recruiting EZH2 at the Nrf2 promoter to inhibit the Nrf2 expression in lipopolysaccharide (LPS)-treated microglia cells (77), and it enhances the NF-κB response by competitively binding to *miR-199b* during acute spinal cord injury (78). Similar to *MALAT1*, *JINR1* regulates transcription and acts as a ceRNA for *miR-216b-5p* and *miR-1-3p* to promote the expression of GRP78 and DDX5. GRP78 facilitates viral entry and replication of JEV, ZIKV, DENV, and other viruses in their host cells (54–56). We have previously shown that GRP78 acts downstream of *JINR1 and* RBM10 to promote JEV, WNV, and DENV replication in SH-SY5Y cells (38). Multiple viruses hijack DDX5 to facilitate or inhibit viral replication, and DDX5 acts as a positive regulator of JEV replication by binding to the JEV 3′ UTR region (53, 79, 80). We have previously shown that *JINR1* activates the expression of genes involved in ER stress and neuroinflammation during viral infection by promoting the binding of p65 to their promoters (38). Interestingly, both DDX5 and GRP78 are also known to regulate the NF-κB transcriptional activity (81, 82). Our findings suggest that *JINR1* is a pivotal pro-viral factor involved in JEV/WNV infection that uses multiple mechanisms to promote viral infection and neuronal cell death.

## Data Availability

The supplementary figures and tables are accessible on Figshare at https://doi.org/10.6084/m9.figshare.28701278.v1 (83).
